# Nucleotide sugar dehydratases: Structure, mechanism, substrate specificity, and application potential

**DOI:** 10.1016/j.jbc.2022.101809

**Published:** 2022-03-07

**Authors:** Ulrike Vogel, Koen Beerens, Tom Desmet

**Affiliations:** Centre for Synthetic Biology (CSB) – Unit for Biocatalysis and Enzyme Engineering, Faculty of Bioscience Engineering, Ghent University, Gent, Belgium

**Keywords:** dehydratase, nucleotide sugars, deoxy sugars, amino sugars, biocatalysis, 4k6d, 4-keto-6-deoxy, Abe, abequose, ASAT, aspartame aminotransferases, dTDP, deoxythymidine diphosphate, GalE, Glc 4-epimerase, GDP, guanosine diphosphate, Glc, glucose, GlcNAc, *N*-acetylglucosamine, GMD, GDP-Man 4,6-dehydratase, l-Fuc, l-fucose, LPS, lipopolysaccharide, l-Rha, l-rhamnose, Man, mannose, NDP, nucleoside diphosphate, NS, nucleotide sugar, NS-SDR, NS active short chain dehydrogenase/reductase, Par, paratose, PLP, pyridoxal phosphate, PMP, pyridoxamine 5′-phosphate, Pse, pseudaminic, QuiNAc, *N*-acetyl-quinovosamine, Tyv, tyvelose, UDP, uridine diphosphate, UGND, UDP-GlcNAc 4,6-dehydratase, WTA, wall teichoic acid

## Abstract

Nucleotide sugar (NS) dehydratases play a central role in the biosynthesis of deoxy and amino sugars, which are involved in a variety of biological functions in all domains of life. Bacteria are true masters of deoxy sugar biosynthesis as they can produce a wide range of highly specialized monosaccharides. Indeed, deoxy and amino sugars play important roles in the virulence of gram-positive and gram-negative pathogenic species and are additionally involved in the biosynthesis of diverse macrolide antibiotics. The biosynthesis of deoxy sugars relies on the activity of NS dehydratases, which can be subdivided into three groups based on their structure and reaction mechanism. The best-characterized NS dehydratases are the 4,6-dehydratases that, together with the 5,6-dehydratases, belong to the NS-short-chain dehydrogenase/reductase superfamily. The other two groups are the less abundant 2,3-dehydratases that belong to the Nudix hydrolase superfamily and 3-dehydratases, which are related to aspartame aminotransferases. 4,6-Dehydratases catalyze the first step in all deoxy sugar biosynthesis pathways, converting nucleoside diphosphate hexoses to nucleoside diphosphate-4-keto-6-deoxy hexoses, which in turn are further deoxygenated by the 2,3- and 3-dehydratases to form dideoxy and trideoxy sugars. In this review, we give an overview of the NS dehydratases focusing on the comparison of their structure and reaction mechanisms, thereby highlighting common features, and investigating differences between closely related members of the same superfamilies.

Dehydratases (EC 4.2.1.x) are a group of lyase enzymes that catalyze the breakage of a carbon–oxygen bond, leading to unsaturated products *via* the removal of oxygen and hydrogen (in the form of water) from organic compounds. There are over 150 different dehydratase enzymes (https://enzyme.expasy.org/EC/4.2.1.-) active on a plethora of different substrates. In this review, we will only focus on the dehydratases that are active on nucleotide sugars (NS). These NS dehydratases are involved in the biosynthesis of deoxy and/or amino sugars (1), which will be further explained in the article. Based on their fold and catalytic mechanisms, the NS active dehydratases can be subdivided into three groups: the NS active short-chain dehydrogenase/reductase (NS-SDR)–like (2, 3), the Nudix hydrolase superfamily–like (4, 5, 6), and the aspartame aminotransferase (ASAT)–related dehydratases (1, 7). The majority of NS dehydratases belongs to the SDR superfamily and has been classified in the extended SDR family (2). The NS-SDR superfamily members include three groups based on their product selectivity, namely, 4,6-dehydratases (either C5-retaining or C5-inverting) and the 5,6-dehydratases (3). Several 2,3-dehydratases have been identified in different antibiotic biosynthesis pathways. EvaA from *Amycolatopsis orientalis* is the best-characterized 2,3-dehydratase to date and belongs to the Nudix hydrolase superfamily (6). Multiple representatives of the 3-dehydratases have been identified, including E1, ColD, and SpnQ (7, 8, 9). They are related to ASATs and catalyze the deoxygenation of C3, but each uses a slightly different reaction mechanism (7, 8, 9).

**Figure 1:**
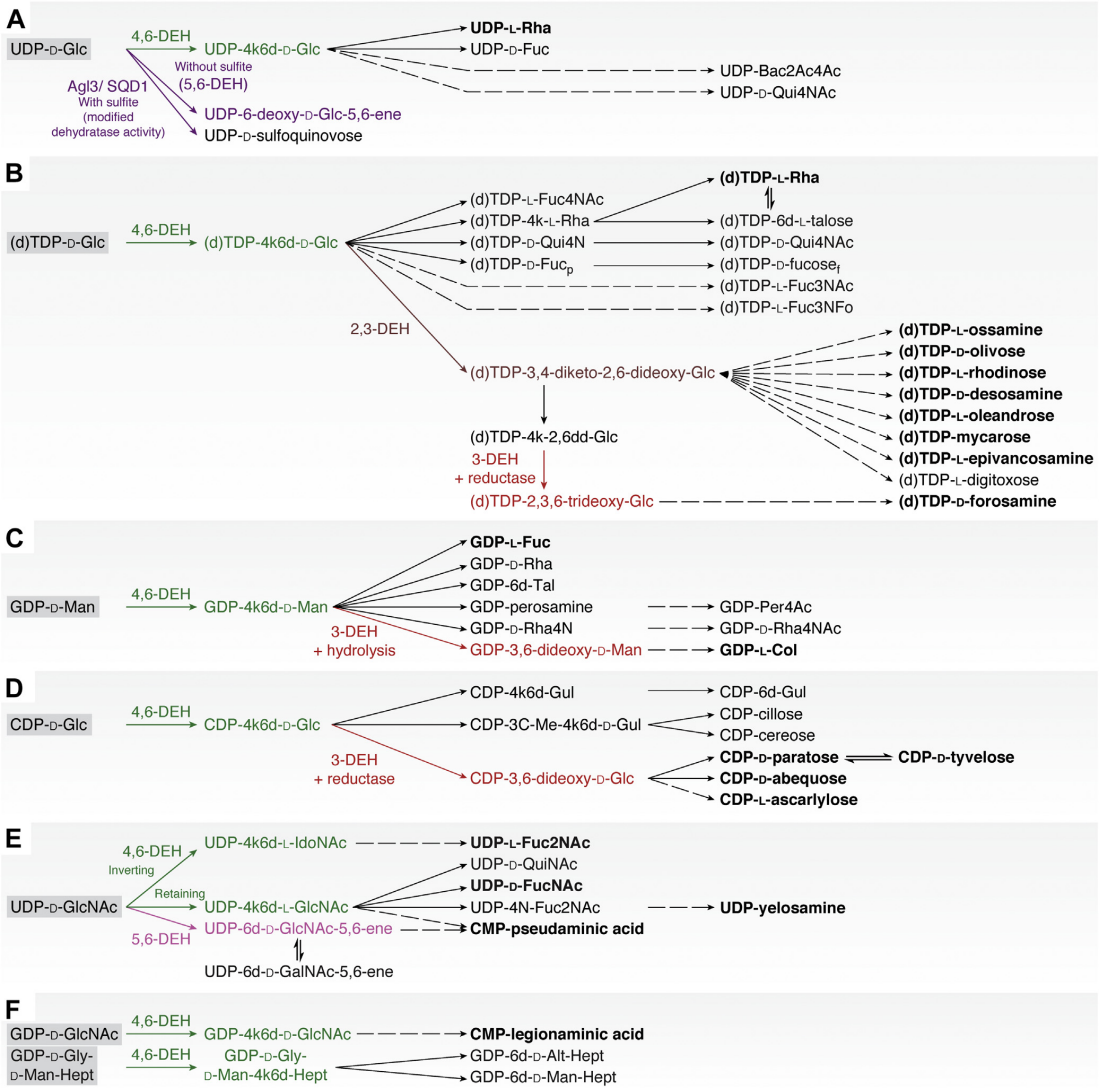
**Bacteria are true masters of deoxy sugar biosynthesis.** They can synthesize an enormous number of different deoxy and amino sugars starting from (*A*) UDP-Glucose, (*B*) (d)TDP-Glc, (*C*) GDP-Man, (*D*) CDP-Glc, (*E*) UDP-GlcNAc, and (*F*) GDP-GlcNAc. The first step in most deoxy sugar biosynthesis pathways is catalyzed by 4,6-dehydratases. The subsequent steps can vary considerably depending on the pathway product. The examples given in the text are highlighted in *bold*. Please note that this is not a complete overview of all deoxy and amino sugars but rather a selection to underline the important roles that NS dehydratases play in the biosynthesis of rare monosaccharides. Reactions catalyzed by 4,6 dehydratases are highlighted in *green*, by 5,6-dehydratases in *pink*, by 3-dehydratases in *red*, and by 2,3-dehydratases in *brown*. (DEH = dehydratases, *solid line arrows* = one catalysis step, *dashed arrow* = multiple catalysis steps). GDP, guanosine dipheosphate; Glc, glucose; GlcNAc, *N*-acetylglucosamine; TDP, thymidine diphosphate; UDP, uridine diphosphate.

**Figure 2:**
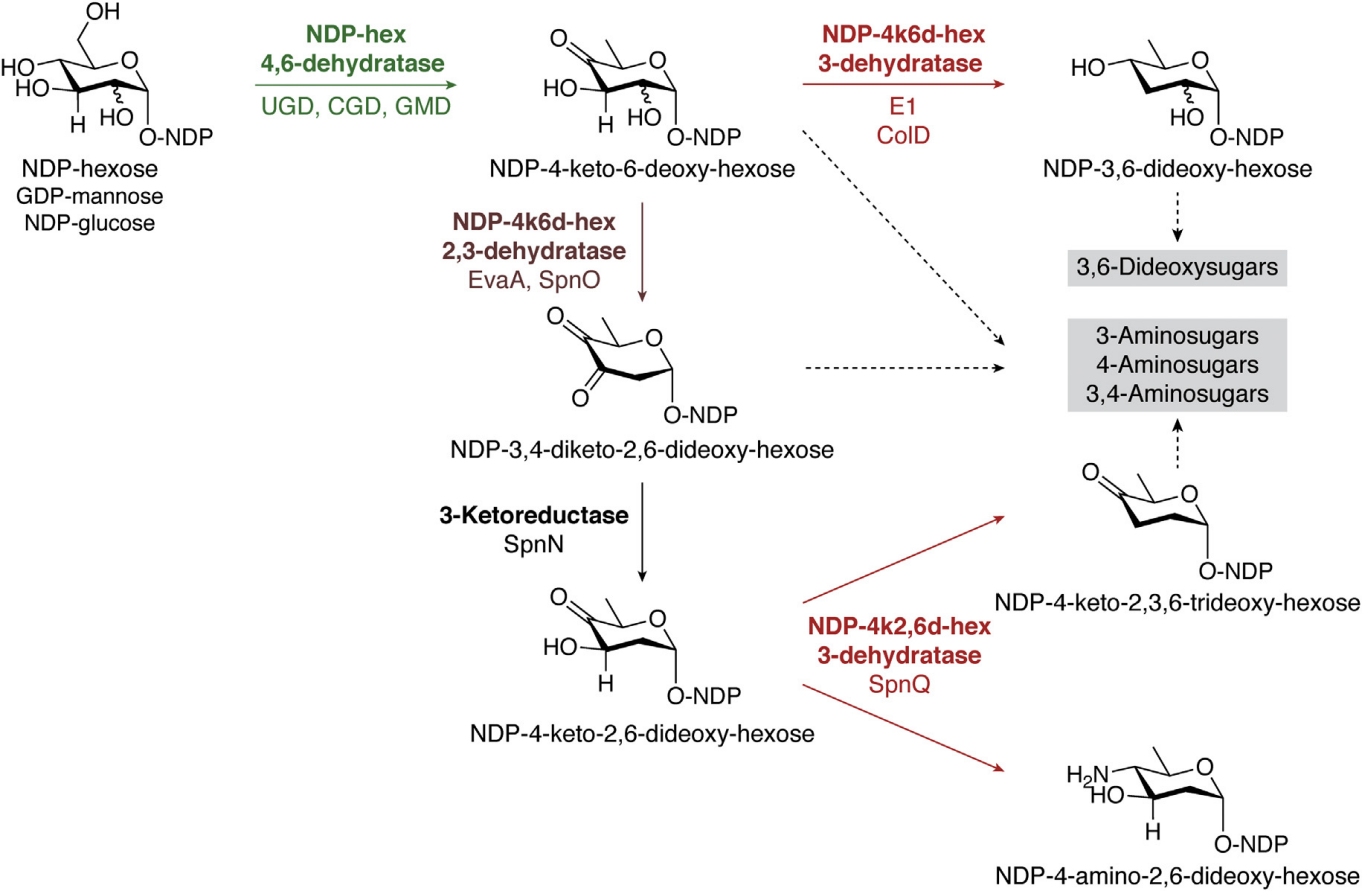
**The different NS dehydratases are involved in the biosynthesis of rare deoxy and amino sugars.** Starting from NDP-glucose and GDP-mannose 4,6-dehydratases catalyze the deoxygenation at C6 and the formation of a keto-functionality at C4. The NDP-4k6d-hexose can in turn be converted by 2,3- and 3-dehydratases which catalyze the subsequent deoxygenation at C2 and/or C3. Depending on the position of the keto-functionality, different aminotransferases can transform the deoxy products into a variety of amino sugars. The position of the keto-group can be altered by 3,4-ketoisomerases. 4k6d, 4-keto-6-deoxy; GDP, guanosine diphosphate; NDP, nucleoside diphosphate; NS, nucleotide sugar.

This review aims to give the reader an overview of the different NS dehydratases. We set the scene by shortly discussing the biosynthesis routes of deoxy sugars in which dehydratases are of key importance, thus highlighting their biological importance and significance in finding new antimicrobial drugs. Afterward, we focus on the NS dehydratases’ structures and mechanisms, as well as their substrate specificities. Herein, we aim to underline common features of the enzymes and emphasize differences among them. We will first focus on the largest group (NS-SDR-type dehydratases) and afterward move on to the less abundant NS 2,3-dehydratase and NS 3-dehydratases. Finally, we end with a future perspective on their application potential.

## Dehydratases are key enzymes for deoxy and amino sugar biosynthesis, conserved in all branches of life

Deoxy and amino sugars are mostly synthesized from the prevalent nucleotide hexoses, nucleoside diphosphate (NDP)–glucose (NDP-Glc), guanosine diphosphate (GDP)–mannose (GDP-Man), and uridine diphosphate (UDP)–*N*-acetylglucosamine (UDP-GlcNAc) (Fig. 1, *A*–*E*) by undergoing several chemical reactions. In most cases, the first step is a 4,6-dehydration followed by (a combination of) epimerization, reduction, ketoisomerization, additional dehydration steps, and aminotransferase reactions, yielding a plethora of NDP-bound special deoxy and amino sugars (Fig. 1) (10). The activity of most aminotransferases requires the presence of a keto-group on the sugar ring (which is introduced through a dehydration step), thus connecting both biosynthesis routes (1). To avoid becoming encyclopedic, we will only highlight a small portion of the examples. Nonetheless, to show their ubiquitous presence, we aimed at providing examples from the different domains of life.

### Two most common deoxy sugars: l-fucose and l-rhamnose

Not surprisingly, the pathways leading to the two most common deoxy sugars, namely l-fucose (l-Fuc) and l-rhamnose (l-Rha) (Fig. 1, *A*–*C*), are highly conserved in all domains of life using homologs of the same enzymes. l-Rha can only be produced by bacteria, fungi, plants, and large DNA viruses, whereas l-Fuc is also synthesized by complex eukaryote-like mammals (10, 11, 12, 13, 14, 15, 16). For example, l-Fuc can be found as a constituent of Lewis blood group antigens and several human milk oligosaccharides and also in bacterial lipopolysaccharide (LPS) layer and plant *N*-glycan structures (14, 17, 18, 19, 20). l-Rha, on the other hand, is less abundant in animals but widely present in plants and bacteria. In plants l-Rha can be found in the primary and secondary cell wall in the sugar polymer pectin and also as a sugar moiety on glycosylated flavonoids (11, 18, 21, 22).

The biosynthesis of these two common deoxy sugars relies on an analogous two-step pathway, where GDP-Man and deoxythymidine diphosphate (dTDP)/UDP-Glc are the starting points, respectively (Fig. 1, *A*–*C*) (11, 14, 15, 23, 24, 25, 26, 27, 28). The first step in both their biosynthesis pathways is performed by a 4,6-dehydratase eliminating the C6-OH and introducing a keto-group at C4, yielding a 4-keto-6-deoxy (4k6d) pathway intermediate. The next steps may differ. The GDP-l-Fuc pathway is conserved throughout all the different domains of life (10, 12, 14, 29, 30), and the GDP-4k6d-Man is converted to GDP-l-Fuc by a one-domain bifunctional 3,5-epimerase/4-reductase also known as GDP-fucose synthase (Fig. 1*C*) (14, 15, 31).

The l-Rha biosynthesis is less conserved and can differ depending on the organism. NDP-l-Rha can be synthesized from either UDP-Glc or dTDP-Glc (Fig. 1, *A* and *B*). Plants, viruses, and fungi are reported to utilize UDP-Glc as the starting substrate, whereas bacteria and some microalgae prefer (deoxy)thymidine as a nucleotide anchor, thus starting from (d)TDP-Glc (11, 16, 23, 24, 25, 26, 27, 28). After the initial 4,6-dehydration step converting NDP-Glc to NDP-4k6d-Glc (16, 28, 32), consecutive 3,5-epimerization and 4-reduction steps are needed to yield NDP-l-Rha. These can be catalyzed by a single bifunctional (but two-domain) enzyme or by two separate enzymes. The latter is the case in bacteria (16, 28, 32, 33), whereas in plants and large DNA viruses, both activities are combined into a single bifunctional enzyme, called UDP-rhamnose synthase (25, 27, 34). In some cases, even trifunctional enzymes have been identified that combine all three activities (4,6-dehydratase, 3,5-epimerase, and the 4-reductase) into one protein to convert UDP-Glc to UDP-l-Rha (26). These (multidomain) bifunctional and trifunctional enzymes have likely arisen from gene fusion and duplication events (24).

Bacteria are true masters of rare deoxy and amino sugar biosynthesis as they have evolved to produce a plethora of highly specialized monosaccharides. To synthesize a wide array of dideoxy, trideoxy, tetradeoxy, and amino sugars, they encode additional 2,3- and 3-dehydratases whose products are consequently converted to extremely versatile and specialized monosaccharides (Figs. 1 and 2). Many of these special sugars end up in the LPS layer and capsule of gram-negative bacteria or in secondary metabolites (*i.e.*, antibiotics).

## Perspective: From biological function to the prospect of application

Deoxy sugars fulfill a wide range of biological functions in different organisms, where they can be found in glycan structures, oligosaccharides, and glycosylated secondary metabolites. In this section we focus on some biological functions in bacteria since this is needed to highlight the NS dehydratases’ application potential. A detailed description of the physiological roles of l-Fuc in mammals and l-Rha in plants can be found elsewhere (11, 14, 16, 35, 36).

In short, we will focus on the role that NS dehydratases play in the search for new antibiotic targets, novel antimicrobial compounds, and diversification of existing antibiotics. Considering the increasing antibiotic resistance of pathogenic bacteria, the search for novel antibiotic agents is ongoing. Owing to the deoxy sugars’ roles in bacterial viability and virulence in both gram-negative and gram-positive bacteria, their biosynthesis pathways have been proposed as new targets for antibiotic design. 4,6-Dehydratases, particularly, have been investigated in that respect due to their early position in all deoxy sugar biosynthesis pathways (33, 37, 38, 39, 40, 41, 42, 43). However, critically speaking, 3- and 2,3-dehydratases could be more interesting antibiotic targets to avoid (unwanted) inhibition of the mammalian/human 4,6-dehydratases. On the other hand, bacterial deoxy sugar biosynthesis may also provide the key to broadening the range of exciting antibiotics by glycodiversification.

### Rare deoxy sugars can be found in the glycolipid lipopolysaccharides of gram-negative bacteria

dTDP-l-Rha, GDP-Fuc, and other deoxy and amino sugars, such as UDP-*N*-acetyl-quinovosamine (UDP-QuiNAc), cytidine tyvelose (CDP-Tyv), UDP-*N,N′*-diacetylbacillosamine, pseudaminic (Pse) acid, CDP-abequose (CDP-Abe), and CDP-paratose (CDP-Par) (Fig. 2), are the precursors of structural elements of the LPS layer of gram-negative bacteria. Examples of such pathogenic species include *Yersinia pseudotuberculosis*, *Salmonella enterica* (serovar Typhimurium), *Escherichia coli*, *Shigella* sp., *Pseudomonas aeruginosa*, *Campylobacter jejuni*, and *Helicobacter pylori* (44, 45, 46, 47, 48, 49, 50).

In gram-negative bacteria the cell wall consists of the inner membrane, the peptidoglycan layer, and an asymmetrical outer membrane (51, 52). Only the outer leaflet of the outer membrane contains glycolipid LPSs, whose structure consists of three components: lipid A, the core oligosaccharides, and the O-antigen (52, 53). Lipid A anchors the LPS structure into the outer membrane. A linker composed of 3-deoxy-α-D-manno-octulosonic acid modified with heptulose monosaccharides and hexose oligosaccharides connects the O-antigen to lipid A (18, 52). The O-antigen is a polysaccharide that is composed of an oligosaccharide repeat-unit with 2 to 6 monosaccharides (18, 52). The O-antigens are highly variable between different bacterial species and strains (54). Their heterogeneity between different strains allows the differentiation of *E. coli*, *Salmonella*, and other bacteria serotypes. O-antigen heterogeneity is achieved through the incorporation of different monosaccharides (including the mentioned rare deoxy sugars) and with variable glyosidic linkages between those (rare) monosaccharides (46, 55, 56).

In general, the LPS functions as an important barrier between the cell and its environment, protecting it against many toxins. Bacterial O-antigens, particularly, play important roles in host–bacteria interactions and virulence on different levels (18). O-antigens can protect the bacterial cell from complement recognition, cell lysis, and phagocytic engulfment and also allow pathogenic bacteria to attach to the epithelial cell surface and to mimic host-specific antigens to evade the immune system (18).

Several rare deoxy sugars, such as *N*-acetyl-QuiNAc, Tyv, diacetylbacillosamine, Abe, and Par that are found in bacterial O-antigens but are absent in human glycan structures, have shown to impact the infection rate of those bacteria. The absence of the rare sugars in the O-antigens can result in lower infection rates (57, 58, 59). Therefore, the enzymes involved in biosynthesis pathways of rare deoxy/amino sugars have become prospective targets for novel small-molecule enzyme inhibitors. While other steps in the LPS biosynthesis have successfully been targeted by novel small-molecule inhibitors, little progress has been reported in the design of inhibitors targeting the biosynthesis pathways of the LPS NS building blocks. For more information on the precise LPS biosynthesis, modification, and potential targets of inhibitors, we would like to refer to reviews by Whitfield and Trent (2014) and Simpson and Trent (2019) that cover these topics in great detail (60, 61).

### Pse acid is essential for flagella assembly in *H. pylori* and *C. jejuni*

In *H. pylori* and *C. jejuni*, flagella are also considered virulence factors as motility is required for host colonization. The modification of flagellin with Pse acid is necessary for the correct assembly of the flagella in both species (62, 63, 64, 65). Pse acid is derived from UDP-GlcNAc through a six-step biosynthetic pathway starting with a 5-inverting 4,6-dehydratase (Fig. 2*E*) (38, 66, 67). When the flagellin glycosylation pathway is inhibited, the flagella of *H. pylori* and *C. jejuni* are truncated, leading to a reduced plaque formation on endothelial HeLa cell cultures (63, 64, 68). A Cj1121c (an aminotransferase downstream of the 4,6-dehydratase in the Pse acid pathway in *C. jejuni*) knockout strain was tested on CaCo-2 cell cultures (cell line of human colorectal adenocarcinoma cells) and live chicks (65). The knockout strain also produced truncated flagella (65). Despite showing better attachment to the CaCo-2 cells (than the wild-type strain), the Cj1121c knockout strain failed at evading the CaCo-2 cells and was unable to colonize the chicken intestine (65). The inhibition of the Pse acid biosynthesis pathway may thus be an efficient target to fight *H. pylori* and *C. jejuni* infections.

### l-Rha as structurally important element of gram-positive and mycobacterial cell walls

In gram-positive bacteria the cell membrane is surrounded by a thick peptidoglycan layer. The precise composition of the peptidoglycan layer is versatile but generally consists of polysaccharide chains that are cross-linked by short peptides (69). Wall teichoic acid (WTA) molecules, capsule polysaccharides, and proteins are anchored to the peptidoglycan layer (70, 71, 72). WTAs intertwine and extend outside with the peptidoglycan layer and play an important role in the physiology and virulence of gram-positive bacteria (70, 72). Typically, WTAs are covalently linked to the polysaccharide chain of the peptidoglycan layer through a phosphodiester bond (70, 72). Although the structure of WTAs differs among different gram-positive species, they generally are composed of a linkage unit (two hexoses) and polymer consisting of alditol-phosphate units (also called polyol units), whose hydroxyl groups can be substituted with different molecules (for example, d-alanyl or d-glucosyl groups) (70, 72). It was observed that some pathogenic species including *Streptococcus suis*, *Streptococcus pyogenes*, *Streptococcus mutants*, and *Bacillus anthracis* synthesize a rhamnose-rich polysaccharide with similar functions as WTAs (73, 74, 75, 76). A comprehensive review of l-Rha-containing cell wall polysaccharides in gram-positive bacteria has been previously provided by Mistou *et al.* (2016) (76). dTDP-l-Rha is a precursor of the various l-Rha-containing cell wall polysaccharides. When the dTDP-l-Rha biosynthesis pathway was inhibited, *S. suis*, *S. pyogenes*, *S. mutants*, and *B. anthracis* showed lower viability presumably due to lower cell wall stability, which in turn had a negative impact on the bacteria’s virulence (32, 33, 77, 78). l-Rha biosynthesis enzymes are not encoded by mammalian cells and have therefore been proposed as target for novel antibiotic drugs. 5-(4-chlorophenyl)-2-furoic acid has been shown to successfully inhibit the dTDP-l-Rha biosynthesis in different *Streptococcus* strains and also in *Mycobacterium tuberculosis* (33).

The cell wall of *M. tuberculosis* is constructed differently from gram-positive bacteria. Here, the peptidoglycan layer is covalently attached to a polysaccharide layer consisting of a galactofuran layer that is in turn attached to the branched arabinofuran layer, which is functionalized with mycolic acid chains (79, 80). An l-Rha(α1→α3)-GlcNAc-linker unit connects the peptidoglycan layer to the galactofuran layer (79, 80). dTDP-l-Rha is the precursor molecule of that linker unit. It was shown that the 4,6-dehydratase in the dTDP-l-Rha biosynthesis pathway (RmlB, Table 1) is essential for the growth of *Mycobacteria* because strains where RmlB was knocked out did not grow (81). Furthermore, 11 compounds that inhibit either RmlB or other enzymes further along in the l-Rha biosynthesis pathway have been identified (82). Important to note here is that those reported compounds are structural analogs of RmlB’s dTDP-Glc substrate, either resembling the nucleotide anchor or the sugar moiety. It remains therefore important to investigate whether these compounds might also interfere with the enzymes involved in mammalian l-Fuc biosynthesis, which would ideally be avoided to prevent unwanted inhibition of the host’s enzymes.

### Rare deoxy sugars are an important structural element of macrolide antibiotics

**Figure 3:**
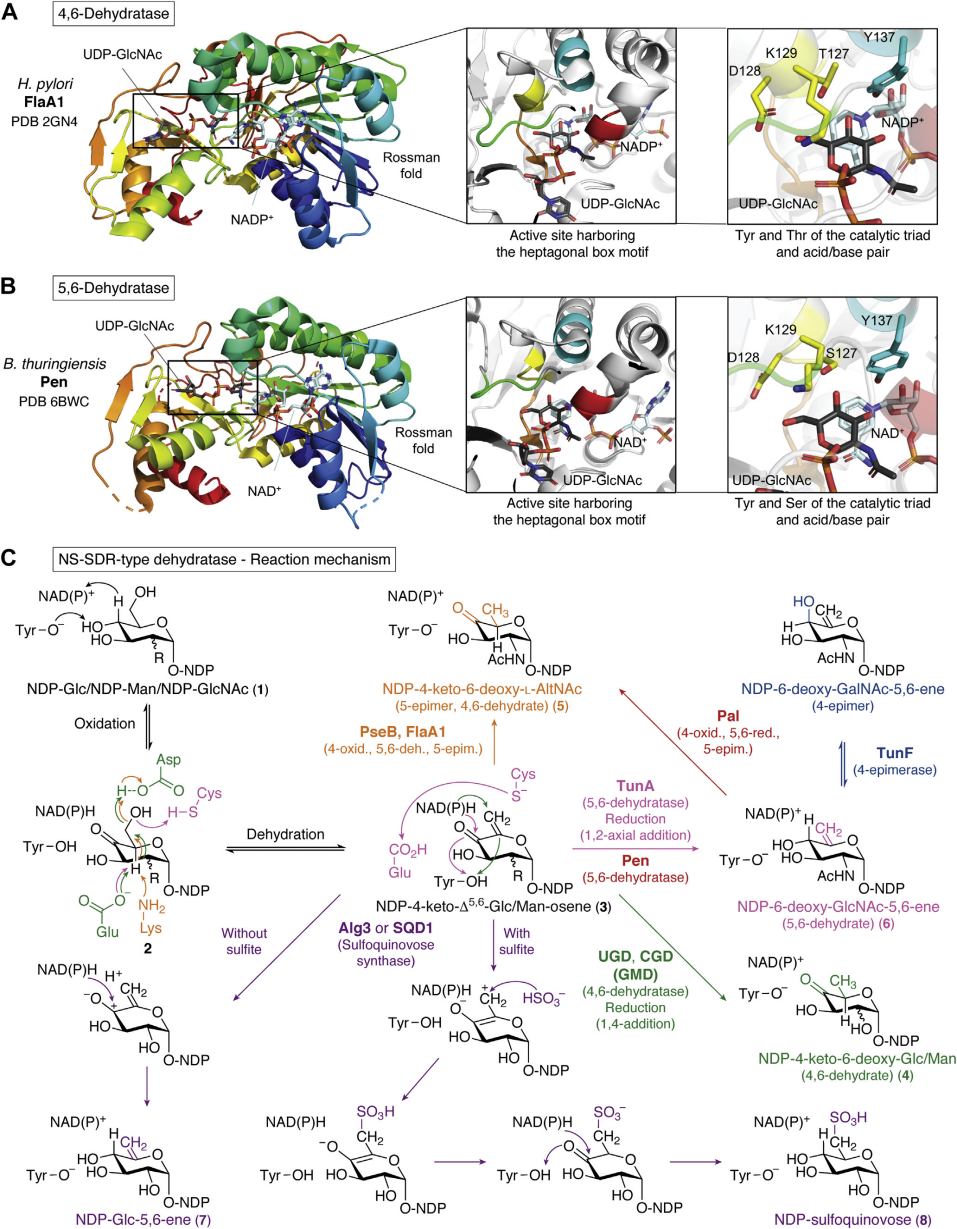
**Structure and active site architecture of the NS-SDR-type dehydratases and the comparison of their different reactivities.** Two representative crystal structures were chosen, namely one 4,6-dehydratase (FlaA1) and one 5,6-dehydratase (Pen). The N-terminal end is colored blue, and the C-terminal end is colored *red*. *A*, crystal structure of an NS 4,6-dehydratase monomer from *Helicobacter pylori* (FlaA1, PDB-ID: 2GN4) with UDP-GlcNAc and NADP^+^ bound in the active site. *B*, crystal structure of an NS 5,6-dehydratase monomer from *Bacillus thuringiensis* (Pen, PDB-ID: 6BWC, note that S128 and N129 were substituted with the wild-type residues D128 and K129 using the PyMOL mutagenesis tool) with UDP-GlcNAc and NAD^+^ bound in the active site. In both (*A*) and (*B*), the active site regions were colored according to the heptagonal box model by Da Costa *et al.* (2021) (3). The Tyr (*cyan*) and Thr/Ser (*yellow*) of the catalytic triad ([ST]x_n_Yx_3_K, K not displayed) as well as the acid/base pairs (*yellow*) involved in catalysis are highlighted in both enzymes. *C*, overview of the different NS-SDR-type dehydratase reactivities: 4,6-dehydratase (retaining or inverting), 5,6-dehydratases and UDP-sulfoquinovose synthase, displaying 5,6-dehydratase activity in the absence of sulfite. GlcNAc, *N*-acetylglucosamine; NS-SDR, NS active short chain dehydrogenase/reductase; UDP, uridine diphosphate.

On the other hand, dehydratases are also involved in the natural antibiotic biosynthesis pathways. Some *Streptomyces* species and *Saccharopolyspora spinosa* synthesize macrolide antibiotics that belong to the polyketide antibiotics and are mostly active against gram-positive bacteria (83). Macrolide antibiotics inhibit protein biosynthesis by targeting the ribosomal 50S subunit (83). They are constructed from macrocyclic lactone rings of different sizes, to which one or multiple deoxy or amino sugars are attached (83). Such deoxy and amino sugars include l-oassamine, d-olivose, l-rhodinose, d-desosamine, mycarose, oleandrose, forosamine, and epivancosamine, which are derived from NDP-Glc or GDP-Man (9, 84, 85, 86, 87, 88, 89) (Figs. 2 and 3). Typically, the sugar units of macrolide antibiotics have undergone deoxygenation by 4,6-; 2,3-; and 3-dehydratases in at least two positions delivering dideoxy, trideoxy, and tetradeoxy sugars that can subsequently be converted to amino sugars (9, 84, 85, 86, 87, 88, 89) (Fig. 2). As antibiotic resistance is on the rise, the search for modifications of existing macrolide antibiotics focusses on the substitution of functional groups on the lactone ring as well as the alteration of the carbon chain length forming the macrocyclic ring (83). Another approach is the alteration, exchange, or removal of the attached sugar moieties (83, 90). This taps into the concept of glycodiversification (also called glycorandomization), which describes the idea of increasing the diversity of natural compounds by altering their carbohydrate structures and thereby modulating their biological and pharmacokinetic activities, which is a promising strategy for drug development (10, 91, 92, 93).

The problem of multidrug-resistant bacteria and the concept of glycodiversification have driven efforts to identify and characterize enzymes in specialty sugar biosynthesis. Since deoxy and amino sugars play integral roles in both objectives, we can clearly state that future research focused on the further understanding as well as the application of NS dehydratases will benefit these fields.

### Bacterial dideoxy/trideoxy/tetradeoxy sugar biosynthesis requires at least two different NS dehydratases

l-Colitose (l-Col, Fig. 1*C*) and Par/Tyv/Abe (Fig. 1*D*) are dideoxy sugars found in the LPSs of some *E. coli*, *Y. pseudotuberculosis*, *S. enterica*, and/or *Vibrio cholerae* strains (45, 94, 95, 96). Like in the GDP-l-Fuc and NDP-l-Rha biosynthesis, the first step is performed by a 4,6-dehydratase, after which a 3-dehydratase (ColD in the colitose pathways and E1 for Par/Tyv/Abe biosynthesis) removes the hydroxyl at C3 (Fig. 2) (94, 97). Finally, additional catalytic steps are required (*e.g.*, reduction and/or epimerization) to obtain the final special sugars (94, 97). These deoxy sugars are then incorporated into polysaccharide structures and translocated to the cell surface to form the bacterial O-antigens as part of the LPS (45, 53, 94, 95, 96).

Moreover, several 2,3-dehydratases have been identified in different macrolide antibiotics biosynthesis pathways of various *Streptomyces* species and *S. spinosa* (9, 84, 85, 86, 88, 89). 2,3-Dehydratases convert the product of 4,6-dehydratases by removing the C2-hydroxyl group and introducing an additional keto-group at C3, thereby yielding a NDP-3,4-diketo-3,6-deoxyhexose intermediate, which undergoes further reductions, dehydration, epimerization, and transamination reactions, yielding trideoxy, tetradeoxy, as well as amino sugars (Fig. 2) (6, 9).

### Different dehydratase reactivities on nucleoside diphosphate-N-acetylglucosamine

Four different dehydratases are active in pathways starting from UDP-GlcNAc or GDP-GlcNAc (Fig. 1, *E* and *F*), including retaining 4,6-dehydratase involved in the biosynthesis of UDP-QuiNAc, yelosamine, and UDP-*N*-acetyl-l-fucosamine ( found in *Bacillus cereus* and *P. aeruginosa*, respectively) as well as in the biosynthesis of CMP-legionaminic acid (in *C. jejuni* and *Legionella pneumophila*) (98, 99, 100, 101, 102). The other dehydratases active on UDP-GlcNAc are the 5-inverting 4,6-dehydratases (performing an additional epimerization at C5) involved in the biosynthesis of Pse acid (in *H. pylori* and *C. jejuni*) (38) and also the 5,6-dehydratase (TunA in *Streptomyces lysosuperificus*) that yields UDP-6-deoxy-5,6-glycosene as an intermediate in the pathway for biosynthesis of tunicamycins (archetypal nucleoside antibiotics targeting the bacterial peptidoglycan biosynthesis and eukaryotic protein N-glycosylation) (103, 104).

As underlined by the aforementioned examples, dehydratases active on NS are present in all domains of life, where they play a key role in the biosynthetic pathways of bacterial (*e.g.*, LPS, capsule, and other cell surface polysaccharides), mammalian (*e.g.*, ABO antigens), and plant (*e.g.*, cell wall polymers) glycans and saccharides (*e.g.*, human milk oligosaccharide) and also for antibiotics and other secondary metabolite synthesis. A detailed description of these different pathways and other enzymes (*e.g.*, reductases, ketoisomerases, aminotransferases, and acetyltransferases) involved in the deoxy and amino sugar biosynthesis, however, lies outside of the scope of this review.

### Nucleotide sugar dehydratases and natural evolution

Although only a limited number of substrates (NDP-Glc, GDP-Man, and UDP/GDP-GlcNAc) are used by nature to expand the diversity of carbohydrate structures (and thus functions) in a process referred to as natural glycodiversification (105), more than 340 distinct appended carbohydrates (small selection shown in Fig. 1) have been found in natural products (106). The NS dehydratases highlighted here play a key role in the existence of many of these special carbohydrates due to the importance of both the deoxygenation and the introduced keto-functionality for consecutive chemical transformations (additional deoxygenation, amination, epimerization, reduction, *etc.*). Considering their position early in the biosynthesis pathways of unusual sugars, the wider diversity in specificities, and their broader phylogenetic distribution, one could speculate that the 4,6-dehydratases may have been among the first NS dehydratases to have come to existence. Further discussion on the evolution of NS dehydratases (or existence of the plethora of rare sugars), however, would become too speculative (certainly without additional ancestral analysis) and is thus out of the scope of our review. In the following sections, we will describe the application potential, structure, catalytic mechanisms, and substrate specificities of the presently know NS dehydratases.

## (Nucleotide sugar-) short-chain dehydrogenase/reductase-type dehydratases

### Classification & structure

The NS-SDR-type dehydratases display a two-domain structure, which is typical for the extended SDR enzymes (2). The C-terminal domain takes on the Rossmann fold (alternating α-helices and β-sheets, which fold into a layer of β-sheets that is surrounded by multiple α-helices (107)) that contains the Glycine motif (Gx_2–3_Gx_1–2_G) involved in NAD^+^–cofactor binding and the catalytic dyad (Yx_3_K, Fig. 3, *A* and *B*) (2, 3). The N-terminal domain contains the NS-binding active site. The catalytic dyad can be extended to a triad ([ST]x_n_Yx_3_K) with the conserved Ser/Thr in the NS-binding active site (Fig. 3, *A* and *B* and Table 1) whose importance during catalysis has been shown previously (3). For a detailed description of the extended SDR-fold structure, we refer to reviews by Kavanagh *et al.* (2) and Da Costa *et al.* (3).

**Table 1:** Mechanistic similarities demonstrate the evolutionary connection between GalE and the NS-SDR dehydratases

Dehydratase	Abbr.	Other names	Catalytic triad (incl. acid/base)	Acid	Base	References
4-epimerase						
UDP-Glc 4-epimerase	GalE		[ST] x_n_Yx_3_K	-	-	(3, 109, 128, 129)
4,6-dehydratases (C5-retaining)						
dTDP/UDP-Glc 4,6-dehydratase	UGD, dTGD	DesIV, R141	[ST]DEx_n_Yx_3_K	Asp	Glu	(3, 42, 130)
CDP-Glc 4,6-dehydratase	CGD		[ST]DKx_n_Yx_3_K	Asp	Lys	(3, 131, 132)
GDP-Man 4,6-dehydratase	GMD		[ST]SEx_n_Yx_3_K	Glu	Water (+Glu)	(3, 115)
UDP-GlcNAc 4,6-dehydratase (ret.)	UGNDr	RmlB, Pdeg	[ST]DEx_n_Yx_3_K	Asp	Glu	(66, 98, 133)
UDP-GlcNAc 4,6-dehydratase (PglF)	WbpM, PglF		TDKx_n_Mx_3_K	Thr/Asp	Lys	(41, 67, 116)
4,6-dehydratases (C5-inverting)						
UDP-GlcNAc 4,6-dehydratase (inv.)	UGNDi	PseB (Cj1293), FlaA1	[ST]DKx_n_Yx_3_K	Asp	Lys	(38, 41, 134, 135, 136)
5,6-dehydratases						
UDP-GlcNAc 5,6-dehydratase	TunA		TCEx_n_Yx_3_K	Cys	Glu	(103, 104)
UDP-GlcNAc 5,6-dehydratase	Pen		[ST]DKx_n_Yx_3_K	Asp	Lys	(62, 117)
UDP-Sulfoquinovose synthase, in the absence of sulfite	SQD1, Agl3		[ST]MGx_n_Yx_3_K	*unknown*	*unknown*	(118, 120)

Here, we will focus on the different active site structures that define the enzymes’ substrate and product specificities. Within the NS-SDR enzymes, different signature sequence fingerprint motifs can be observed for each reactivity and thus also for the dehydratase specificities (3). These fingerprints have been summarized as the heptagonal box motif of the NS-SDR family. The seven walls of the heptagonal box surround the nucleotide sugar in the active site and determine the enzymes’ substrate and product specificity (Fig. 3, *A* and *B*) (3). Based on these fingerprints, five different groups of NS dehydratases can be differentiated: four groups of 4,6-dehydratases with different NS substrates (*i.e.*, dTDP/UDP-glucose, CDP-glucose, GDP-mannose, and UDP-GlcNAc/-Glc, Fig. 1, *A*–*E*) and the fifth group of 5,6-dehydratases active on UDP-GlcNAc (Fig. 3 and Table 1) (3, 108). The 4,6-dehydratases in turn can be split up into two subgroups. Some 4,6-dehydratases are capable of an additional epimerization at C5 (C5-inverting), which differentiates them from the more common C5-retaining 4,6-dehydratases (38, 41).

The most important heptagonal box walls for all NS-SDR enzymes are the cyan and yellow walls, containing the catalytic dyad (Yx_3_K) and the additional acid/base residue(s) needed for dehydratase activity, respectively (3). It is interesting to notice that different additional acid/base residues achieve the same 4,6-dehydration, namely an Asp–Lys couple for CDP-Glc 4,6-dehydratase, an Asp–Glu couple for both dTDP-Glc 4,6-dehydratase, and UDP-GlcNAc 4,6-dehydratase (UGND), or a single Glu in case of GDP-Man 4,6-dehydratase (GMD) (Table 1) (3).

### Mechanism

#### General mechanism: Nucleoside diphosphate-Glc 4,6-dehydratase (retaining)

This section aims to give a generalized overview of the mechanisms involved in the conversion of NDP-glucose (NDP-Glc 1) to NDP-4k6d-Glc 4. The retaining NDP-Glc dehydratases are dTDP-glucose 4,6-dehydratase, CDP-glucose 4,6-dehydratase, and UGND_r_ (Table 1). The reaction mechanism of NS-SDR 4,6-dehydratases shares common steps with other NS-SDR enzymes, of which the UDP-Glc 4-epimerase (GalE) is best understood (109).

Similar to GalE (109), 4,6-dehydratases are expected to undergo a conformational change upon substrate binding (110, 111). The conformational change is likely induced through binding of the nucleotide anchor (42), which causes the active site to be covered by an alpha helix (110, 111).

A key feature of all NS-SDR enzymes is the first catalytic step, the oxidation of the glycosyl C4 (3). A hydride is transferred between the NS substrate and enzyme-bound NAD(P)^+^ cofactor (3, 109, 112). The different 4,6-dehydratase reactions are also initiated through a C4-oxidation of their NS substrate by the NAD^+^ cofactor and the conserved tyrosine (Yx_3_K) (Fig. 3, *A* and *B*) (3, 42, 110). The nicotinamide ring of the NAD^+^ cofactor abstracts a hydride from the glycosyl C4 (110). Simultaneously, the catalytic tyrosine acts as the catalytic base and abstracts a proton from the C4-hydroxyl group (C4-OH) to complete the first step (oxidation) and yield the typical 4-keto-intermediate 2 (Fig. 3*C*, black) (42). This 4-keto-intermediate is likely stabilized by Thr/Ser present in the yellow wall (Fig. 3, *A* and *B*) (42). The importance of this Thr/Ser has been shown previously, and the catalytic dyad is therefore extended to a triad ([ST]x_n_Yx_3_K) in the NS-SDR enzymes (3).

The same can be observed in the UDP-GalE (109) where Tyr also acts as catalytic acid to assist the deprotonation of C4-OH (Fig. 3, black) (3). In GalE the 4-keto-intermediate undergoes a rotation followed by the transfer of the hydride back to the glycosyl C4 to complete the 4-epimerization (109). In 4,6-dehydratases the subsequent reaction is more complex and additional catalytic residues are required. The initial C4-oxidation is followed by the dehydration and reduction of the glycosyl C6 (28, 111, 113). The additional catalytic acid and base accomplish the elimination of water (dehydration) and generate a 4-keto-5,6-glycosene intermediate 3 (28, 42) (Fig. 3, black). Interestingly, the catalytic acid and base responsible for this dehydration are distinct for the different dehydratase specificities (Table 1) (3). For the dehydration of C6, the catalytic acid of the yellow wall (typically: Asp, Fig. 3, *A* and *B*) protonates C6-OH, thereby eliminating water from C6 (42). Subsequently, the neighboring catalytic base (typically: Glu or Lys) abstracts a proton from the C5, generating the NDP-4-keto-5,6-glucosene intermediate 3. The last catalytic step is the reduction of the glycosyl C6. A hydride will be transferred back to C6 from the cofactor, while the proton is transferred back to C5 by the catalytic base (Glu or Lys) (114). The original conformation at C5 is retained (retaining activity) (28). The final NDP-4k6d-hexose 4 product is formed and leaves the active site (Fig. 3, green) (114).

In the following paragraphs, we will discuss the details and peculiarities of the different dehydratase specificities that divert from this more classical retaining NDP-Glc 4,6-dehydratase mechanism.

#### Dehydration mechanism in GDP-Man 4,6-dehydratases only uses one additional catalytic residue

The GMD is also a retaining 4,6-dehydratase, yielding GDP-4k6d-Man 4 from GDP-Man 1 (115). Overall, the oxidation of C4 and the dehydration and reduction of C6 are catalyzed similarly as in other NS-SDR 4,6-dehydratases. However, this enzyme displays a more parsimonious dehydration mechanism in comparison to the NDP-Glc 4,6-dehydratases, as was shown by the Nidetzky group (115). GMD contains only one additional catalytic residue (Glu) that serves as catalytic acid and indirectly transfers a proton to C5 *via* a water molecule in the active site (Fig. 3, *green*) (115).

#### C5-inverting UDP-GlcNAc 4,6-dehydratase

Rather interestingly, some UDP-GlcNAc 4,6-dehydratases are known to possess a C5-inverting activity (UGND_i_), yielding UDP-4k6d-l-IdoNAc 5 (sometimes also called UDP-4k6d-l-AltNAc 5, Fig. 3, *orange*) (38, 40). In this case, the proton is transferred back at the opposite side of the C5 (and the hydride to C6), resulting in the additional C5-epimerization step (38, 40). To date, however, it could not be determined which active site residues may be involved in this additional 5-epimerization (38, 40). It could merely be shown that the proton that is transferred back to C5 during the epimerization is derived from water inside the active site (38).

#### UDP-GlcNAc 4,6-dehydratases without the catalytic tyrosine (Mx_3_K)

WbpM from *P. aeruginosa* and PglF from *C. jejuni* are other special UDP-GlcNAc 4,6-dehydratase variants. WbpM is particularly interesting as its structure can be subdivided into two regions: the C-terminal domains with the typical NS-SDR dehydratase characteristics and an N-terminal region that acts as membrane anchor and targets WbpM to the inner membrane of *P. aeruginosa* (116). It was shown that the two WbpM regions could be split up and that the C-terminal region is fully functional as 4,6-dehydratase when linked to a His-tag instead (41, 116).

In the C-terminal 4,6-dehydratase region of WbpM and in PglF, the typical catalytic Tyr has been substituted by a methionine (Met) (41, 67, 116). It was demonstrated that both WbpM and PglF are still active without the catalytic Tyr in the cyan wall (Table 1) (41, 67, 116).

The M438Y variant of WbpM was created and compared to the FlaA1 Y141M variant (an inverting UDP-GlcNac 4,6-dehydratase, Table 1) (41). Interestingly, both variants retained some activity. Contrary to the author’s expectations, the *K*_*M*_ and *k*_cat_ values of WbpM M438Y decreased (compared to the wild-type WbpM), despite the variant containing the typical NS-SDR catalytic diad (Yx_3_K) (41). From the analysis of both variants, it was hypothesized that the Met would still act as catalytic base with a higher local p*K*_*a*_ in the active site (than Tyr of FlaA1) (41). However, in a later study on the crystal structure of PglF, the new catalytic mechanism for the 4,6-dehydratases containing the Mx_3_K dyad was proposed (67). It was found that the Met does not act as a general base as previously suggested based on the WbpM studies (41, 67). Instead, it was shown that the combination of Thr395 and Asp396 from the yellow wall removes the proton from the C4-OH (C4-oxidation) (67). The neighboring Lys (Lys397) is involved in the dehydration and reprotonation of C6 (67).

#### 5,6-Dehydratase yields an exo-glycal product

TunA from *S. lysosuperificus* was the first reported UDP-GlcNAc 5,6-dehydratase (103, 104). The 5,6-dehydratase activity was later also observed in two more enzymes, namely in Pen from *Bacillus thuringiensis*, also a UDP-GlcNAc 5,6-dehydratase (117), and in the UDP-sulfoquinovose synthase in the absence of sulfite (converts UDP-Glc) (118).

The first two steps during catalysis of TunA are identical to those in 4,6-dehydratases. The final reduction step, however, proceeds differently: the proton is transferred back to C4 and the hydride to the keto-functionality (103). This altered reduction results in the formation of an exo-glycal product, NDP-6-deoxy-5,6-glycosene 6, where a double bond is formed outside the ring between C5 and C6 (103) (Fig. 3, pink). When comparing TunA to 4,6-dehydratases, the conserved Asp/Glu acid/base couple has been substituted by Cys/Glu. The active-site Cys likely allows the altered reactivity of TunA (103). Although expected to act as a general acid, the cysteine's higher p*K*_*a*_ may prevent unwanted protonation. The Cys may also provide more favorable hydrophobic interactions with the formed C=C double bond (103).

Quite interestingly, Pen’s catalytic triad and acid/base residues (TDKx_n_Yx_3_K) differ from those of TunA (TCEx_n_Yx_3_K) and correspond to those in the UDP-GlcNAc 4,6-dehydratases (TDKx_n_Yx_3_K, Table 1 and Fig. 3, *red*) (62, 117). Considering the enzyme’s active site architecture, the catalytic mechanism likely shares common steps with the UDP-GlcNAc 5-inverting 4,6-dehydratases (62). The oxidation of C4 and water elimination at C6 are proposed to apply the same mechanism (62, 117).

Strikingly, there seems to be one common difference between both UDP-GlcNAc 5,6-dehydratases (TunA and Pen) compared to the inverting UDP-GlcNAc 4,6-dehydratases (*i.e.*, PseB [formerly Cj1293] from *C. jejuni*) (62). In TunA and Pen, the nicotinamide ring of the NAD(P)^+^ cofactor is angled toward C4 of the GlcNAc moiety, whereas it is parallel to the glycosyl ring in PseB (62, 117). The differently angled nicotinamide ring causes an increased distance to the glycosyl C6 that might prevent reprotonation at C6 in the 5,6-dehydratases, which is the essential step to complete 4,6-dehydration (62).

#### Modified dehydratase mechanism for UDP-sulfoquinovose synthase results in 5,6-dehydration in the absence of sulfite

The UDP-sulfoquinovose synthase (Agl3 (119) or SQD1 (120)) normally converts UDP-Glc and sulfite to UDP-sulfoquinovose (Fig. 3, *purple*). This activated form of sulfoquinovose is required for its incorporation into glycoconjugates or glycoprotein N-glycans (118, 119, 120). Recent structure determination and point mutation studies of Agl3 allowed in-depth mechanistic analyses (118). Its complex reaction cycle is suggested to be a modified dehydratase mechanism that includes the following steps: oxidation, dehydration, enolization, sulfite addition, and reprotonation (118). Different reaction products can be formed dependent on the type and the amount of substrate. In the absence of sulfite, this enzyme acts as a 5,6-dehydratase yielding UDP-Glc-5,6-ene 7 instead of the UDP-sulfoquinovose 8 if sulfite is present (118) (Fig. 3, *purple*).

## 2,3-Dehydratases from the Nudix superfamily

### Classification & structure

Multiple dTDP-Glc 2,3-dehydratases have been identified and are involved in the production of highly deoxygenated dTDP-hexoses during antibiotic biosynthesis (84, 85, 86, 87, 88, 89, 121). The identified dTDP-Glc 2,3-dehydratases include among others Gra27, TylX3, OleV, OssT, and UrdS, which are involved in granaticin, tylosin, oleandomycin, ossamycin, and urdamycin biosynthesis, respectively, and occur in different *Streptomyces* species (84, 85, 86, 87, 88). Other examples are SpnO from *Saccharopolyspora spinosa* (spinosyn biosynthesis) (121) and EvaA from *A. orientalis* (chloroeremomycin biosynthesis) (89).

**Figure 4:**
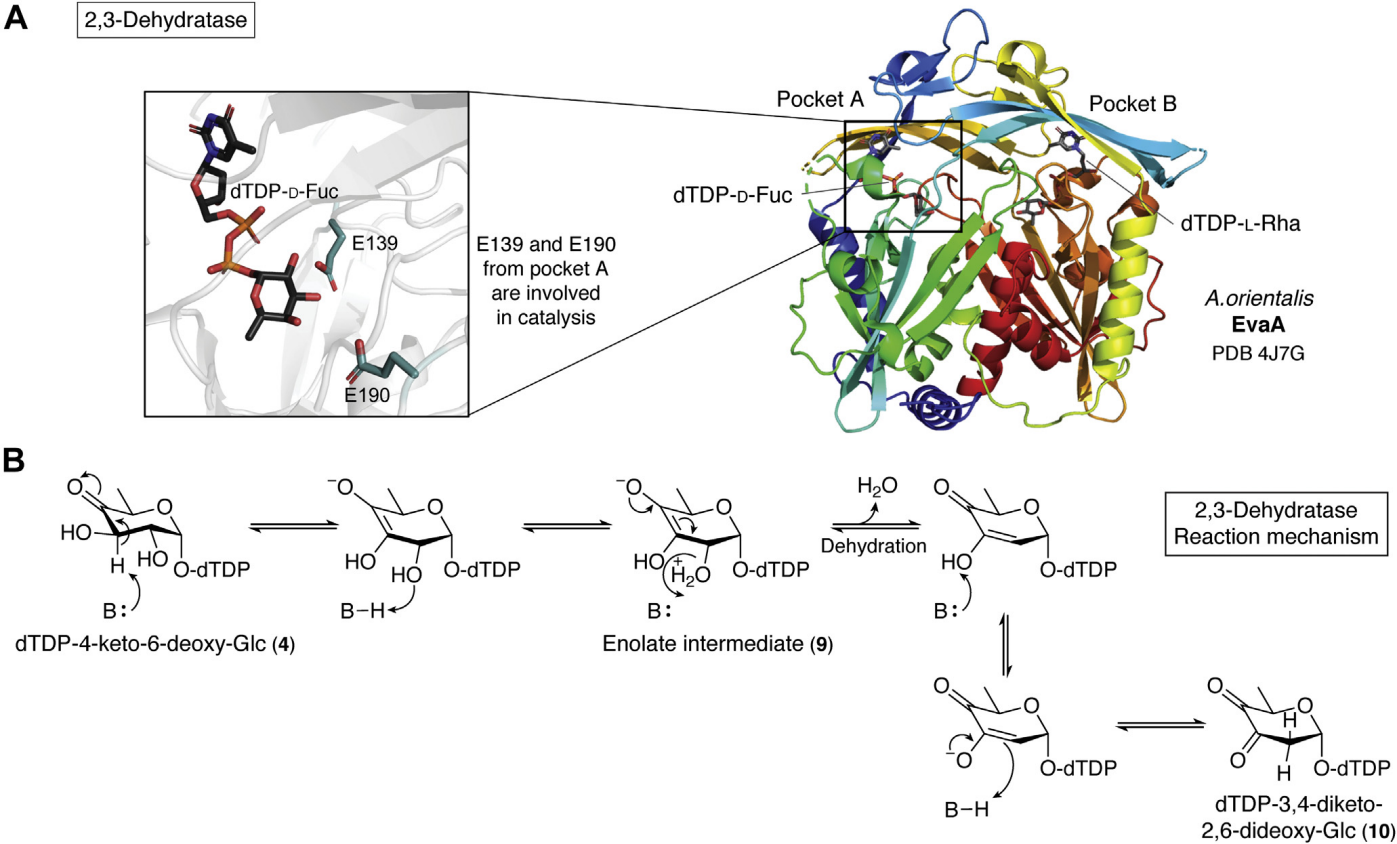
**Structure and mechanism of the Nudix hydrolase superfamily dehydratases.***A*, crystal structure of NS 2,3-dehydratase monomer from *Amycolatopsis orientalis* (EvaA) harboring dTDP-d-fucose in the active site (pocket A) and dTDP-l-rhamnose in the second remnant substrate-binding pocket B (PDB-ID: 4J7G). Inside pocket A, the residues E139 and E190 are highlighted since they are likely involved in catalysis of EvaA. *B*, the active site base from EvaA abstracts a proton from C3 that is delivered to C2-OH, causing the elimination of water at C2. Afterward, the catalytic base abstracts a proton from C3-OH and delivers it back to C2 yielding the dTDP-3,4-diketo-2,6-dideoxy hexose. (Figure adapted from Kubiak *et al.*, 2013). dTDP, deoxythymidine diphosphate.

Despite the increasing number of reported NS 2,3-dehydratase homologs, only EvaA is well characterized, and we will thus focus on EvaA in the following section. EvaA belongs to the Nudix hydrolase superfamily, which stands for “NDP linked to some other moiety, X” (6, 122). Members of the Nudix hydrolase superfamily are often referred to as “housekeeping genes” as they are involved in the degradation of toxic metabolites (4, 122). Enzymes of the Nudix hydrolase superfamily are characterized by a 23-amino-acid signature motif which is located in a helix–loop–helix structural motif (Gx_5_Ex_7_REUxKEx_2_U, U = I, L or V) (4). EvaA forms a dimer, of which each subunit folds into two domains with two separate NS-binding pockets A and B (Fig. 4*A*) (6). Pocket A functions as the active site, while pocket B is an inactive leftover from a gene duplication event (6).

### Mechanism

A catalytic mechanism for this 2,3-dehydratase was proposed based on an analogous two-step reaction for the formation of dTDP-2,6-dideoxyhexoses (88). This two-step reaction is a part of the granaticin, oleandomycin, and tylosin biosynthesis pathways that also start from dTDP-4k6d-Glc 4 (Figs. 2 and 4) (6, 88, 123). Despite the lack of amino acid residues within 3.5 Å of the substrate analog in the EvaA crystal structure, it was suggested that an active site catalytic base abstracts a proton from C3, yielding an enolate intermediate 9 (6). In the following step, the proton would be delivered to C2-OH, which causes the elimination of water at C2 (6). Afterward, the catalytic base abstracts a proton from C3-OH and delivers it back to C2 to yield the dTDP-3,4-diketo-2,6-dideoxy hexose 10 (Fig. 4*B*) (6). Mutagenesis of the active site residues E139 and E190 from pocket A (Fig. 4*A*) led to a complete loss of activity, indicating that both residues may be involved in catalysis (6). Mutation of E352 and D353 in pocket B retained activity of the enzyme, indicating that only pocket A was participating in catalysis (6).

## 3-Dehydratases: Relatives of aminotransferases

### Classification & structure

Different NS 3-dehydratases with three different substrate preferences have been identified in the biosynthetic pathways of complex bacterial deoxy and amino sugars. E1 from *Y. pseudotuberculosis* (7, 124), ColD from different pathogenic gram-negative bacteria (8, 94, 96, 125), and SpnQ from *S. spinosa* and *S. pogona* (9, 121) are well characterized. Other NS 3-dehydratases have been identified, but not further characterized *in vitro*, in the biosynthesis pathways of urdamycin and ossamycin (84, 85).

**Figure 5:**
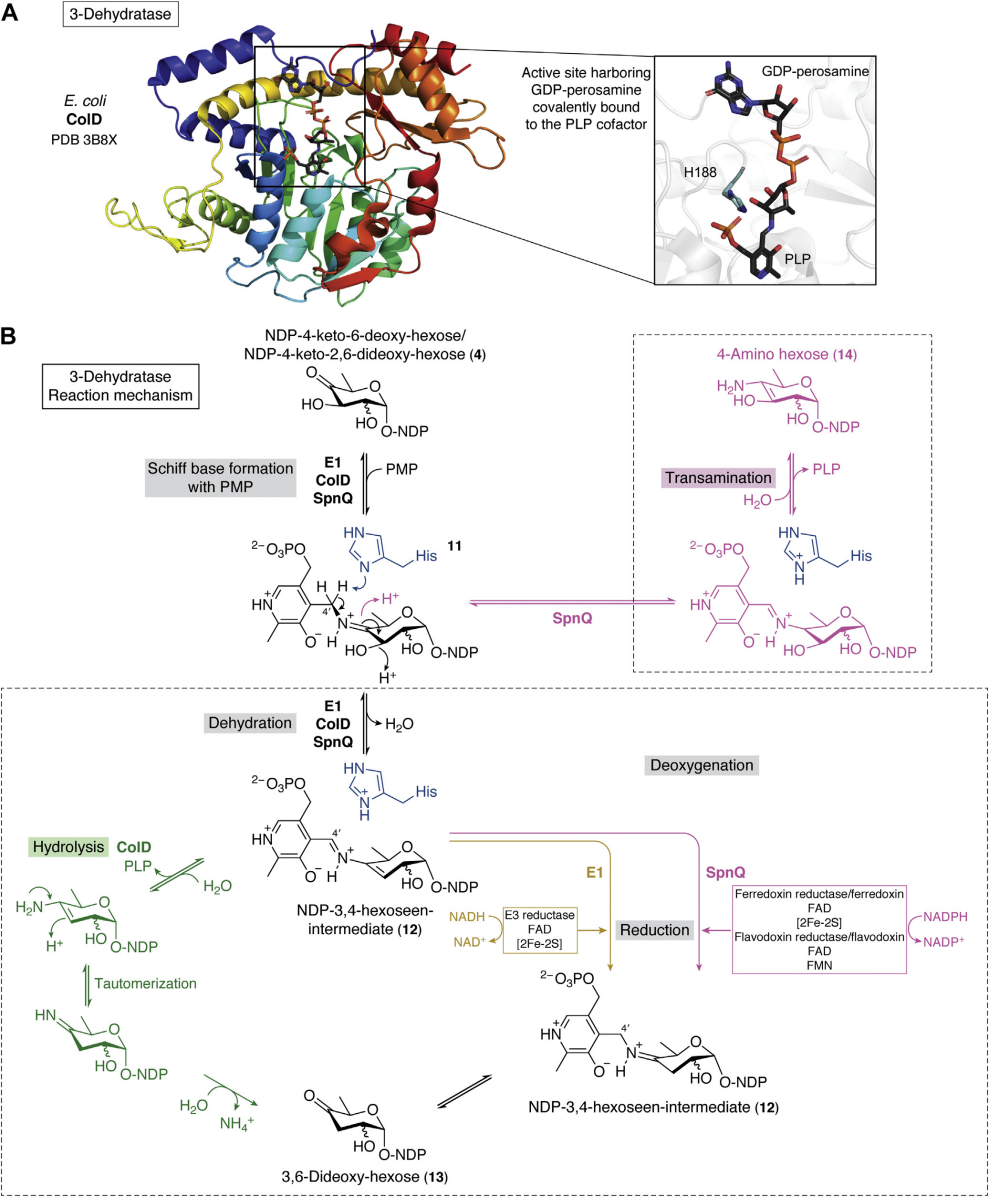
**Structure and mechanism of the 3-dehydratase.***A*, crystal structure of NS 3-dehydratase monomer from *Escherichia coli* (ColD). GDP-perosamine is covalently bound to the pyridoxal phosphate (PLP) cofactor and is trapped in the structure (PDB-ID: 3B8X, please note that K188 was mutated back to H188 using the PyMOL mutagenesis tool in the display of the active site). *B*, reaction mechanisms of the different 3-dehydratases. The catalyzed reaction of 3-dehydratases starts with the formation of a Schiff base. E1, ColD, and SpnQ form an NDP-3,4-hexoseen-intermediate 12. Subsequently, the pathways split, and the following steps are catalyzed differently by the enzymes. It can be differentiated between a ColD-like and an E1/SpnQ-like pathway. In the ColD-like pathway, the intermediate is hydrolyzed. In the E1/SpnQ-like pathway, a reductase is used to regenerate the cofactor and release the product. Additionally, SpnQ can perform a transamination reaction when sufficient PLP and glutamate are added to the reaction mix. (Figure adapted from Hong *et al.*, 2006 & 2008 (9, 121)). NDP, nucleoside diphosphate; NS, nucleotide sugar.

E1, ColD, and SpnQ are homologs of ASATs, which are illustrated by typical ASAT properties in their protein structures and reaction mechanisms (7, 125). Fittingly, E1 has been titled the “crossroads of dehydration, amino transfer, and epimerization” by Smith *et al.* (7). The crystal structures of E1 and ColD have been solved at high resolutions (1.9 Å and 1.8 Å, respectively, Fig. 5*A*) (8, 96, 124, 125). NS 3-dehydratases are dimeric proteins that take on a mixed β-strand fold that is surrounded by α-helices, which strongly resembles the overall fold of ASATs (7, 124, 125). The number of β-sheets varies depending on the enzyme (seven β-strand in E1, eight β-strand in ColD, Fig. 5*A*). The large buried surface area harbors the active site that is mainly constructed by one subunit and complemented with a loop from the second subunit (124, 125). The dipole moment from one helix points its positive end toward the pyridoxamine 5′-phosphate (PMP) cofactor and helps to bind it in the structure (124, 125). E1 and SpnQ additionally coordinated an iron–sulfur cluster [2Fe-2S] that is absent in ColD (9, 124, 125, 126). Both enzymes harbor a [2Fe-2S]-binding motif in their sequence that is characterized by the presence of four cysteines surrounding the PMP cofactor (Fig. 5*A*) (9, 124, 126). The most important difference compared to ASATs is the substitution of the conserved catalytic lysine with a histidine inside of the active site (Fig. 5*A*) (9, 124, 125). The catalytic histidine acts as catalytic base and acid throughout catalysis and likely initiates the reaction through proton abstraction from the cofactor that leads to the expulsion of the C3-OH (125).

### Mechanism

E1 and ColD catalyze the C3-deoxygenation of CDP-4k6d-Glc and GDP-4k6d-Man 4, respectively, yielding NDP-4-keto-3,6-dideoxy-hexose 13 as the product (94, 124). SpnQ catalyzes the formation of dTDP-4-keto-2,3,6-trideoxy-Glc 13 by eliminating the C3-OH from dTDP-4-keto-2,6-dideoxy-Glc 4 (9). In all three enzymes, the reaction starts analogously with the formation of a Schiff-base 11 between the PMP cofactor and their NDP-4k6d-hexose/NDP-4-keto-2,6-dideoxy-hexose substrate (9, 124, 125). Subsequently, a proton is abstracted from C4 of PMP by the catalytic histidine, which leads to the expulsion of the C3-OH and the formation of an NDP-3,4-hexoseen-intermediate 12 (Fig. 5*B*) (9, 124, 125). The following steps for the reduction of the intermediate differ between E1, ColD, and SpnQ.

In ColD the 3,4-aminomannoseen-intermediate-cofactor-complex 12 is hydrolyzed yielding pyridoxal phosphate (PLP) and an enamine sugar (125). The amino group at C4 is eliminated by tautomerization followed by hydrolysis generating the final GDP-4-keto-2,6-dideoxy-mannose product 13 and NH_4_^+^ (Fig. 5, *green*) (125). A transamination reaction with glutamate as an amino donor regenerates the PMP cofactor (125).

In E1, the CDP-3,4-glucoseen intermediate 12 is reduced by a two-step electron transfer cascade that involves a second [2Fe-2S]-cluster containing enzyme called E3 (Fig. 5, yellow) (124). The NADH cofactor from E3 transfers an electron to E3’s flavin adenine dinucleotidecofactor to start the reaction. Two electrons are subsequently transferred between the iron–sulfur clusters from E3 to E1 leading to the reduction of the CDP-3,4-glucoseen intermediate to CDP-4-keto-3,6-deoxy-Glc 13 (7, 124).

In SpnQ the electron transfer may proceed similarly as in E1, with the difference being that ferredoxin/ferredoxin reductase and flavodoxin/flavodoxin reductase pairs are used instead of the E1-specific enzyme E3 (Fig. 5, *purple*) (9). Interestingly, without a reductase present, SpnQ shows an aminotransferase activity when glutamate and PLP are added in sufficient amounts (Fig. 5, *purple*) 14 (9). When ColD and E1 are applied to similar conditions, the transferase activity was not observed (9). In the H220K variant in which the conserved histidine in E1 was substituted with a lysine (as typically conserved in ASATs), however, a transamination reaction occurred (127). But the reaction was rendered “noncatalytic,” as the PLP cofactor could not be regenerated for subsequent reaction cycles, possibly due to disturbed cofactor binding in the H220K variant (127).

## Conclusion

Although the concept of enzymatic glycodiversification of natural products has first been described about 2 decades ago, we are still far from reaching that goal (10, 91). Despite well-characterized representatives of each NS dehydratase group and our good understanding of the catalytic mechanisms, neither biocatalysis nor efficient biotransformation pathways to produce deoxy/amino sugars on large scale under industrial conditions have been reported yet. More data mining and biochemical characterization of putative enzymes will be necessary to identify thermostable and promiscuous individuals and also to identify possible new dehydratase specificities. Engineering studies could help to further understand the precise catalytic mechanism of existing ones, like the determination of the residues involved and the mechanism of the additional 5-epimerization of the inverting UDP-GlcNAc 4,6-dehydratases. One of the main hurdles in that process is the availability of the NS substrates and the instability of pathway intermediates. Typical biochemical and kinetic parameters (temperature and pH optimum, *K*_*M*_ and *k*_cat_) are the basis of enzymology and biocatalysis. Although the characterization of most 4,6-dehydratases is still relatively easy, as dTDP-/UDP-/GDP-Glc, UDP-GlcNAc, and GDP-Man are commercially available (CDP-Glc, however, is not), enzymes further along in the biosynthesis pathways are, naturally, difficult to characterize due to the lack of substrates. Without the knowledge of the aforementioned biochemical and kinetic parameters of all involved enzymes, it is virtually impossible to optimize a biocatalytic pathway to the point where rare sugar (and/or glycoside) synthesis becomes economically feasible. Metabolic engineering may also be a helpful tool to synthesize rare sugars. The lack of substrates also hinders the development of enzyme inhibitors as new antibiotic agents. High-throughput screening of the enzymes involved in deoxy sugar biosynthesis is usually limited by the substrate cost and also by the lack of appropriate screening methods.

In short, NS dehydratases are a group of intriguing enzymes, found in all domains of life and of major importance for different biological functions. Building on a strong basis of previous fundamental research, an interesting yet challenging future awaits these enzymes, for example, in the fields of applied biological chemistry (*e.g.*, antibiotic synthesis) and systems biocatalysis (*e.g.*, specialty carbohydrate synthesis).

## Conflict of interest

The authors declare that they have no conflicts of interest with the contents of this article.

## References

[1] Romo A.J., Liu H.W. (2011). Mechanisms and structures of vitamin B 6-dependent enzymes involved in deoxy sugar biosynthesis. Biochim. Biophys. Acta.

[2] Kavanagh K.L., Jörnvall H., Persson B., Oppermann U. (2008). Medium- and short-chain dehydrogenase/reductase gene and protein families: The SDR superfamily: Functional and structural diversity within a family of metabolic and regulatory enzymes. Cell. Mol. Life Sci..

[3] Da Costa M., Gevaert O., Van Overtveldt S., Lange J., Joosten H.J., Desmet T., Beerens K. (2021). Structure-function relationships in NDP-sugar active SDR enzymes: Fingerprints for functional annotation and enzyme engineering. Biotechnol. Adv..

[4] McLennan A.G. (2006). The Nudix hydrolase superfamily. Cell. Mol. Life Sci..

[5] Mildvan A.S., Xia Z., Azurmendi H.F., Saraswat V., Legler P.M., Massiah M.A., Gabelli S.B., Bianchet M.A., Kang L.W., Amzel L.M. (2005). Structures and mechanisms of Nudix hydrolases. Arch. Biochem. Biophys..

[6] Kubiak R.L., Thoden J.B., Holden H.M. (2013). Structure of EvaA: A paradigm for sugar 2,3-dehydratases. Biochemistry.

[7] Smith P., Szu P.H., Bui C., Liu H.W., Tsai S.C. (2008). Structure and mutagenic conversion of E1 dehydrase: At the crossroads of dehydration, amino transfer, and epimerization. Biochemistry.

[8] Cook P.D., Holden H.M. (2008). GDP-4-keto-6-deoxy-d-mannose 3-dehydratase, accommodating a sugar substrate in the active site. J. Biol. Chem..

[9] Hong L., Zhao Z., Liu H.W. (2006). Characterization of SpnQ from the spinosyn biosynthetic pathway of *Saccharopolyspora spinosa*: Mechanistic and evolutionary implications for C-3 deoxygenation in deoxysugar biosynthesis. J. Am. Chem. Soc..

[10] Thibodeaux C.J., Melançon C.E., Liu H.W. (2008). Natural-product sugar biosynthesis and enzymatic glycodiversification. Angew. Chem. Int. Ed. Engl..

[11] Jiang N., Dillon F.M., Silva A., Gomez-Cano L., Grotewold E. (2021). Rhamnose in plants - from biosynthesis to diverse functions. Plant Sci..

[12] Piacente F., Gaglianone M., Laugieri M.E., Tonetti M.G. (2015). The autonomous glycosylation of large DNA viruses. Int. J. Mol. Sci..

[13] Schneider M., Al-Shareffi E., Haltiwanger R.S. (2017). Biological functions of fucose in mammals. Glycobiology.

[14] Becker D.J., Lowe J.B. (2003). Fucose: Biosynthesis and biological function in mammals. Glycobiology.

[15] Foster D.W., Ginsburg V. (1961). Biosynthesis of L-fucose by mammalian tissue. Biochim. Biophys. Acta.

[16] Wagstaff B.A., Zorzoli A., Dorfmueller H.C. (2021). NDP-rhamnose biosynthesis and rhamnosyltransferases: Building diverse glycoconjugates in nature. Biochem. J..

[17] Strasser R. (2016). Plant protein glycosylation. Glycobiology.

[18] Lerouge I., Vanderleyden J. (2002). O-antigen structural variation: Mechanisms and possible roles in animal/plant-microbe interactions. FEMS Microbiol. Rev..

[19] Guberman M., Bräutigam M., Seeberger P.H. (2019). Automated glycan assembly of Lewis type I and II oligosaccharide antigens. Chem. Sci..

[20] Bode L. (2015). The functional biology of human milk oligosaccharides. Early Hum. Dev..

[21] Mohnen D. (2008). Pectin structure and biosynthesis. Curr. Opin. Plant Biol..

[22] Bozzo G.G., Unterlander N. (2021). In through the out door: Biochemical mechanisms affecting flavonoid glycoside catabolism in plants. Plant Sci..

[23] Wagstaff B.A., Rejzek M., Kuhaudomlarp S., Hill L., Mascia I., Nepogodiev S.A., Dorfmueller H.C., Field R.A. (2019). Discovery of an RmlC/D fusion protein in the microalga *Prymnesium parvum* and its implications for NDP-L-rhamnose biosynthesis in microalgae. J. Biol. Chem..

[24] Yin Y., Huang J., Gu X., Bar-Peled M., Xu Y. (2011). Evolution of plant nucleotide-sugar interconversion enzymes. PLoS One.

[25] Bockhaus N.J., Ferek J.D., Thoden J.B., Holden H.M. (2020). The high-resolution structure of a UDP-L-rhamnose synthase from Acanthamoeba polyphaga Mimivirus. Protein Sci..

[26] Yin S., Liu M., Kong J.Q. (2016). Functional analyses of OcRhS1 and OcUER1 involved in UDP-L-rhamnose biosynthesis in *Ornithogalum caudatum*. Plant Physiol. Biochem..

[27] Oka T., Nemoto T., Jigami Y. (2007). Functional analysis of Arabidopsis thaliana RHM2/MUM4, a multidomain protein involved in UDP-D-glucose to UDP-L-rhamnose conversion. J. Biol. Chem..

[28] Dong C., Beis K., Giraud M.-F., Blankenfeldt W., Allard S., Major L.L., Kerr I.D., Whitfield C., Naismith J.H. (2003). A structural perspective on the enzymes that convert dTDP-D-glucose into dTDP-L-rhamnose. Biochem. Soc. Trans..

[29] Tonetti M., Zanardi D., Gurnon J.R., Fruscione F., Armirotti A., Damonte G., Sturla L., De Flora A., Van Etten J.L. (2003). *Paramecium bursaria Chlorella* virus 1 encodes two enzymes involved in the biosynthesis of GDP-L-fucose and GDP-D-rhamnose. J. Biol. Chem..

[30] Bonin C.P., Potter I., Vanzin G.F., Reiter W.D. (1997). The MUR1 gene of *Arabidopsis thaliana* encodes an isoform of GDP-d-mannose-4,6-dehydratase, catalyzing the first step in the de novo synthesis of GDP-L-fucose. Proc. Natl. Acad. Sci. U. S. A..

[31] Beerens K., Gevaert O., Desmet T. (2022). GDP-mannose 3,5-epimerase: A view on structure, mechanism, and industrial potential. Front. Mol. Biosci..

[32] Gokey T., Halavaty A.S., Minasov G., Anderson W.F., Kuhn M.L. (2018). Structure of the *Bacillus anthracis* dTDP-L-rhamnose biosynthetic pathway enzyme: dTDP-α-D-glucose 4,6-dehydratase, RfbB. J. Struct. Biol..

[33] van der Beek S.L., Zorzoli A., Çanak E., Chapman R.N., Lucas K., Meyer B.H., Evangelopoulos D., de Carvalho L.P.S., Boons G., Dorfmueller H.C., van Sorge N.M. (2019). Streptococcal dTDP-L-rhamnose biosynthesis enzymes: Functional characterization and lead compound identification. Mol. Microbiol..

[34] Zhao Z., Ren C., Xie L., Xing M., Zhu C., Jin R., Xu C., Sun C., Li X. (2020). Functional analysis of PpRHM1 and PpRHM2 involved in UDP-L-rhamnose biosynthesis in *Prunus persica*. Plant Physiol. Biochem..

[35] Li J., Hsu H.C., Mountz J.D., Allen J.G. (2018). Unmasking fucosylation: From cell adhesion to immune system regulation and diseases. Cell Chem. Biol..

[36] Thomès L., Bojar D. (2021). The role of fucose-containing glycan motifs across taxonomic kingdoms. Front. Mol. Biosci..

[37] Chidwick H.S., Fascione M.A. (2020). Mechanistic and structural studies into the biosynthesis of the bacterial sugar pseudaminic acid (Pse5Ac7Ac). Org. Biomol. Chem..

[38] Morrison J.P., Schoenhofen I.C., Tanner M.E. (2008). Mechanistic studies on PseB of pseudaminic acid biosynthesis: A UDP-*N*-acetylglucosamine 5-inverting 4,6-dehydratase. Bioorg. Chem..

[39] Morrison J., Troutman J., Imperiali B. (2010). Development of a multicomponent kinetic assay of the early enzymes in the *Campylobacter jejuni* N-linked glycosylation pathway. Bioorg. Med. Chem..

[40] Ishiyama N., Creuzenet C., Miller W.L., Demendi M., Anderson E.M., Harauz G., Lam J.S., Berghuis A.M. (2006). Structural studies of FlaA1 from *Helicobacter pylori* reveal the mechanism for inverting 4,6-dehydratase activity. J. Biol. Chem..

[41] Creuzenet C., Urbanic R.V., Lam J.S. (2002). Structure-function studies of two novel UDP-GlcNAc C6 dehydratases/C4 reductases. Variation from the SYK dogma. J. Biol. Chem..

[42] Allard S.T.M., Beis K., Giraud M.F., Hegeman A.D., Gross J.W., Wilmouth R.C., Whitfield C., Graninger M., Messner P., Allen A.G., Maskell D.J., Naismith J.H. (2002). Toward a structural understanding of the dehydratase mechanism. Structure.

[43] Ud-Din A.I.M.S., Roujeinikova A. (2018). Flagellin glycosylation with pseudaminic acid in *Campylobacter* and *Helicobacter*: Prospects for development of novel therapeutics. Cell. Mol. Life Sci..

[44] Verma N.K., Quigley N.B., Reeves P.R. (1988). O-antigen variation in *Salmonella* spp.: rfb gene clusters of three strains. J. Bacteriol..

[45] Kenyon J.J., Cunneen M.M., Reeves P.R. (2017). Genetics and evolution of *Yersinia pseudotuberculosis* O-specific polysaccharides: A novel pattern of O-antigen diversity. FEMS Microbiol. Rev..

[46] Liu B., Furevi A., Perepelov A.V., Guo X., Cao H., Wang Q., Reeves P.R., Knirel Y.A., Wang L., Widmalm G. (2020). Structure and genetics of *Escherichia coli* O-antigens. FEMS Microbiol. Rev..

[47] Liu B., Knirel Y.A., Feng L., Perepelov A.V., Senchenkova S.N., Wang Q., Reeves P.R., Wang L. (2008). Structure and genetics of *Shigella* O antigens. FEMS Microbiol. Rev..

[48] Rocchetta H.L., Burrows L.L., Lam J.S. (1999). Genetics of O-antigen biosynthesis in *Pseudomonas aeruginosa*. Microbiol. Mol. Biol. Rev..

[49] Li H., Liao T., Debowski A.W., Tang H., Nilsson H., Stubbs K.A., Marshall B.J., Benghezal M. (2016). Lipopolysaccharide structure and biosynthesis in *Helicobacter pylori*. Helicobacter.

[50] Karlyshev A., Ketley J., Wren B. (2005). The *Campylobacter jejuni* glycome. FEMS Microbiol. Rev..

[51] Rietschel T.E., Brade L., Schade U., Seydel U., Zähringer U., Brandenburg K., Helander I., Holst O., Kondo S., Kuhn H.M., Lindner B., Röhrscheidt E., Russa R., Labischinski H., Naumann D. (1990). Bacterial lipopolysaccharides: Relationship of structure and conformation to endotoxic activity, serological specificity and biological function. Adv. Exp. Med. Biol..

[52] Mühlradt P.F., Golecki J.R. (1975). Asymmetrical distribution and artifactual reorientation of lipopolysaccharide in the outer membrane bilayer of *Salmonella typhimurium*. Eur. J. Biochem..

[53] Mühlradt P.F., Menzel J., Golecki J.R., Speth V. (1973). Outer membrane of *Salmonella*. Sites of export of newly synthesised lipopolysaccharide on the bacterial surface. Eur. J. Biochem..

[54] Samuel G., Reeves P. (2003). Biosynthesis of O-antigens: Genes and pathways involved in nucleotide sugar precursor synthesis and O-antigen assembly. Carbohydr. Res..

[55] Stenutz R., Weintraub A., Widmalm G. (2006). The structures of *Escherichia coli* O-polysaccharide antigens. FEMS Microbiol. Rev..

[56] Liu B., Knirel Y.A., Feng L., Perepelov A.V., Senchenkova S.N., Reeves P.R., Wang L. (2014). Structural diversity in *Salmonella* O antigens and its genetic basis. FEMS Microbiol. Rev..

[57] Bengoechea J.A., Najdenski H., Skurnik M. (2004). Lipopolysaccharide O antigen status of *Yersinia enterocolitica* O:8 is essential for virulence and absence of O antigen affects the expression of other *Yersinia* virulence factors. Mol. Microbiol..

[58] Gunn J.S., Ryan S.S., Van Velkinburgh J.C., Ernst R.K., Miller S.I. (2000). Genetic and functional analysis of a PmrA-PmrB-regulated locus necessary for lipopolysaccharide modification, antimicrobial peptide resistance, and oral virulence of *Salmonella enterica* serovar typhimurium. Infect. Immun..

[59] Ugalde J.E., Czibener C., Feldman M.F., Ugalde R.A. (2000). Identification and characterization of the *Brucella abortus* phosphoglucomutase gene: Role of lipopolysaccharide in virulence and intracellular multiplication. Infect. Immun..

[60] Whitfield C., Trent M.S. (2014). Biosynthesis and export of bacterial lipopolysaccharides. Annu. Rev. Biochem..

[61] Simpson B.W., Trent M.S. (2019). Pushing the envelope: LPS modifications and their consequences. Nat. Rev. Microbiol..

[62] Delvaux N.A., Thoden J.B., Holden H.M. (2018). Molecular architectures of Pen and Pal: Key enzymes required for CMP-pseudaminic acid biosynthesis in *Bacillus thuringiensis*. Protein Sci..

[63] Thibault P., Logan S.M., Kelly J.F., Brisson J.R., Ewing C.P., Trust T.J., Guerry P. (2001). Identification of the carbohydrate moieties and glycosylation motifs in *Campylobacter jejuni* flagellin. J. Biol. Chem..

[64] Schirm M., Soo E.C., Aubry A.J., Austin J., Thibault P., Logan S.M. (2003). Structural, genetic and functional characterization of the flagellin glycosylation process in *Helicobacter pylori*. Mol. Microbiol..

[65] Vijayakumar S., Merkx-Jacques A., Ratnayake D.B., Gryski I., Obhi R.K., Houle S., Dozois C.M., Creuzenet C. (2006). Cj1121c, a novel UDP-4-keto-6-deoxy-GlcNAc C-4 aminotransferase essential for protein glycosylation and virulence in *Campylobacter jejuni*. J. Biol. Chem..

[66] Schoenhofen I.C., McNally D.J., Vinogradov E., Whitfield D., Young N.M., Dick S., Wakarchuk W.W., Brisson J.-R., Logan S.M. (2006). Functional characterization of dehydratase/aminotransferase pairs from *Helicobacter* and *Campylobacter*. J. Biol. Chem..

[67] Riegert A.S., Thoden J.B., Schoenhofen I.C., Watson D.C., Young N.M., Tipton P.A., Holden H.M. (2017). Structural and biochemical investigation of PglF from *Campylobacter jejuni* reveals a new mechanism for a member of the short chain dehydrogenase/reductase superfamily. Biochemistry.

[68] Ewing C.P., Andreishcheva E., Guerry P. (2009). Functional characterization of flagellin glycosylation in *Campylobacter jejuni* 81-176. J. Bacteriol..

[69] Vollmer W., Blanot D., De Pedro M.A. (2008). Peptidoglycan structure and architecture. FEMS Microbiol. Rev..

[70] Neuhaus F.C., Baddiley J. (2003). A continuum of anionic charge: Structures and functions of D-alanyl-teichoic acids in gram-positive bacteria. Microbiol. Mol. Biol. Rev..

[71] Dramsi S., Magnet S., Davison S., Arthur M. (2008). Covalent attachment of proteins to peptidoglycan. FEMS Microbiol. Rev..

[72] Brown S., Santa Maria J.P., Walker S. (2013). Wall teichoic acids of gram-positive bacteria. Annu. Rev. Microbiol..

[73] Sutcliffe I.C., Black G.W., Harrington D.J. (2008). Bioinformatic insights into the biosynthesis of the group B carbohydrate in *Streptococcus agalactiae*. Microbiology.

[74] Caliot É., Dramsi S., Chapot-Chartier M.-P., Courtin P., Kulakauskas S., Péchoux C., Trieu-Cuot P., Mistou M.-Y. (2012). Role of the group B antigen of Streptococcus agalactiae: A peptidoglycan-anchored polysaccharide involved in cell wall biogenesis. PLoS Pathog..

[75] McCarty M. (1952). The lysis of group A hemolytic streptococci by extracellular enzymes of Streptomyces albus. J. Exp. Med..

[76] Mistou M.Y., Sutcliffe I.C., Van Sorge N.M. (2016). Bacterial glycobiology: Rhamnose-containing cell wall polysaccharides in gram-positive bacteria. FEMS Microbiol. Rev..

[77] van der Beek S.L., Le Breton Y., Ferenbach A.T., Chapman R.N., van Aalten D.M.F., Navratilova I., Boons G.-J., McIver K.S., van Sorge N.M., Dorfmueller H.C. (2015). GacA is essential for group A S treptococcus and defines a new class of monomeric dTDP-4-dehydrorhamnose reductases (RmlD). Mol. Microbiol..

[78] Bischer A.P., Kovacs C.J., Faustoferri R.C., Quivey R.G. (2020). Disruption of L-rhamnose biosynthesis results in severe growth defects in *Streptococcus mutans*. J. Bacteriol..

[79] Brennan P.J., Nikaido H. (1995). The envelope of mycobacteria. Annu. Rev. Biochem..

[80] Brennan P. (2003). Structure, function, and biogenesis of the cell wall of Mycobacterium tuberculosis. Tuberculosis.

[81] Li W., Xin Y., McNeil M.R., Ma Y. (2006). RmlB and rmlC genes are essential for growth of mycobacteria. Biochem. Biophys. Res. Commun..

[82] Ma Y., Stern R.J., Scherman M.S., Vissa V.D., Yan W., Jones V.C., Zhang F., Franzblau S.G., Lewis W.H., McNeil M.R. (2001). Drug targeting *Mycobacterium tuberculosis* cell wall synthesis: Genetics of dTDP-rhamnose synthetic enzymes and development of a Microtiter plate-based screen for inhibitors of conversion of dTDP-glucose to dTDP-rhamnose. Antimicrob. Agents Chemother..

[83] Dinos G.P. (2017). The macrolide antibiotic renaissance. Br. J. Pharmacol..

[84] Bilyk O., Samborskyy M., Leadlay P.F. (2019). The biosynthetic pathway to ossamycin, a macrocyclic polyketide bearing a spiroacetal moiety. PLoS One.

[85] Hoffmeister D., Ichinose K., Domann S., Faust B., Trefzer A., Dräger G., Kirschning A., Fischer C., Künzel E., Bearden D., Rohr J., Bechthold A. (2000). The NDP-sugar co-substrate concentration and the enzyme expression level influence the substrate specificity of glycosyltransferases: Cloning and characterization of deoxysugar biosynthetic genes of the urdamycin biosynthetic gene cluster. Chem. Biol..

[86] Aguirrezabalaga I., Olano C., Allende N., Rodriguez L., Braña A.F., Méndez C., Salas J.A. (2000). Identification and expression of genes involved in biosynthesis of oleandrose and its intermediate olivose in the oleandomycin producer *Streptomyces antibioticus*. Antimicrob. Agents Chemother..

[87] Chen H., Agnihotri G., Guo Z., Que N.L.S., Chen X.H., Liu H.-W. (1999). Biosynthesis of mycarose: Isolation and characterization of enzymes involved in the C-2 deoxygenation. J. Am. Chem. Soc..

[88] Draeger G., Park S.H., Floss H.G. (1999). Mechanism of the 2-deoxygenation step in the biosynthesis of the deoxyhexose moieties of the antibiotics granaticin and oleandomycin. J. Am. Chem. Soc..

[89] Chen H., Thomas M.G., Hubbard B.K., Losey H.C., Walsh C.T., Burkart M.D. (2000). Deoxysugars in glycopeptide antibiotics: Enzymatic synthesis of TDP-L-epivancosamine in chloroeremomycin biosynthesis. Proc. Natl. Acad. Sci. U. S. A..

[90] Agouridas C., Denis A., Auger J.M., Benedetti Y., Bonnefoy A., Bretin F., Chantot J.F., Dussarat A., Fromentin C., D'Ambrières S.G., Lachaud S., Laurin P., Le Martret O., Loyau V., Tessot N. (1998). Synthesis and antibacterial activity of ketolides (6-O-methyl-3-oxoerythromycin derivatives): A new class of antibacterials highly potent against macrolide-resistant and -susceptible respiratory pathogens. J. Med. Chem..

[91] Williams G.J., Gantt R.W., Thorson J.S. (2008). The impact of enzyme engineering upon natural product glycodiversification. Curr. Opin. Chem. Biol..

[92] Langenhan J.M., Griffith B.R., Thorson J.S. (2005). Neoglycorandomization and chemoenzymatic glycorandomization: Two complementary tools for natural product diversification. J. Nat. Prod..

[93] Goel B., Tripathi N., Mukherjee D., Jain S.K. (2021). Glycorandomization: A promising diversification strategy for the drug development. Eur. J. Med. Chem..

[94] Alam J., Beyer N., Liu H.W. (2004). Biosynthesis of colitose: Expression, purification, and mechanistic characterization of GDP-4-keto-6-deoxy-d-mannose-3-dehydrase (ColD) and GDP-L-colitose synthase (ColC). Biochemistry.

[95] Reeves P.R., Cunneen M.M., Liu B., Wang L. (2013). Genetics and evolution of the *Salmonella* galactose-initiated set of O antigens. PLoS One.

[96] Cook P.D., Holden H.M. (2007). A structural study of GDP-4-keto-6-deoxy-d-mannose-3-dehydratase: Caught in the act of geminal diamine formation. Biochemistry.

[97] Hallis T.M., Lei Y., Que N.L.S., Liu H.W. (1998). Mechanistic studies of the biosynthesis of paratose: Purification and characterization of CDP-paratose synthase. Biochemistry.

[98] Hwang S., Aronov A., Bar-Peled M. (2015). The biosynthesis of UDP-d-QuiNAc in *Bacillus cereus* ATCC 14579. PLoS One.

[99] Hulrooney E.F., Poon K.K.H., McNally D.J., Brisson J.R., Lam J.S. (2005). Biosynthesis of UDP-*N*-acetyl-L-fucosamine, a precursor to the biosynthesis of lipopolysaccharide in *Pseudomonas aeruginosa* serotype O11. J. Biol. Chem..

[100] Hwang S., Li Z., Bar-Peled Y., Aronov A., Ericson J., Bar-Peled M. (2014). The biosynthesis of UDP-d-FucNAc-4N-(2)-oxoglutarate (UDP-Yelosamine) in *Bacillus cereus* ATCC 14579: Pat and Pyl, an aminotransferase and an ATP-dependent Grasp protein that ligates 2-oxoglutarate to UDP-4-amino-sugars. J. Biol. Chem..

[101] Schoenhofen I.C., Young N.M., Gilbert M. (2017). Biosynthesis of legionaminic acid and its incorporation into glycoconjugates. Methods Enzymol..

[102] Hassan M.I., Lundgren B.R., Chaumun M., Whitfield D.M., Clark B., Schoenhofen I.C., Boddy C.N. (2016). Total biosynthesis of legionaminic acid, a bacterial sialic acid analogue. Angew. Chem. Int. Ed. Engl..

[103] Wyszynski F.J., Lee S.S., Yabe T., Wang H., Gomez-Escribano J.P., Bibb M.J., Lee S.J., Davies G.J., Davis B.G. (2012). Biosynthesis of the tunicamycin antibiotics proceeds via unique exo-glycal intermediates. Nat. Chem..

[104] Wyszynski F.J., Hesketh A.R., Bibb M.J., Davis B.G. (2010). Dissecting tunicamycin biosynthesis by genome mining: Cloning and heterologous expression of a minimal gene cluster. Chem. Sci..

[105] Thibodeaux C.J., Melançon C.E., Liu H.W. (2007). Unusual sugar biosynthesis and natural product glycodiversification. Nature.

[106] Elshahawi S.I., Shaaban K.A., Kharel M.K., Thorson J.S. (2015). A comprehensive review of glycosylated bacterial natural products. Chem. Soc. Rev..

[107] Shin W.H., Kihara D. (2019). 55 Years of the Rossmann fold. Methods Mol. Biol..

[108] Tiwari P., Singh N., Dixit A., Choudhury D. (2014). Multivariate sequence analysis reveals additional function impacting residues in the SDR superfamily. Proteins.

[109] Beerens K., Soetaert W., Desmet T. (2015). UDP-hexose 4-epimerases: A view on structure, mechanism and substrate specificity. Carbohydr. Res..

[110] Beis K., Allard S.T.M., Hegeman A.D., Murshudov G., Philp D., Naismith J.H. (2003). The structure of NADH in the enzyme dTDP-D-glucose dehydratase (RmlB). J. Am. Chem. Soc..

[111] Hegeman A.D., Gross J.W., Frey P.A. (2001). Probing catalysis by *Escherichia coli* dTDP-glucose-4,6-dehydratase: Identification and preliminary characterization of functional amino acid residues at the active site. Biochemistry.

[112] Borg A.J.E., Beerens K., Pfeiffer M., Desmet T., Nidetzky B. (2021). Stereo-electronic control of reaction selectivity in short-chain dehydrogenases: Decarboxylation, epimerization, and dehydration. Curr. Opin. Chem. Biol..

[113] Allard S.T.M., Giraud M.F., Whitfield C., Graninger M., Messner P., Naismith J.H. (2001). The crystal structure of dTDP-D-glucose 4,6-dehydratase (RmlB) from *Salmonella enterica* serovar Typhimurium, the second enzyme in the dTDP-L-rhamnose pathway. J. Mol. Biol..

[114] Allard S.T.M., Cleland W.W., Holden H.M. (2004). High resolution X-ray structure of dTDP-glucose 4,6-dehydratase from *Streptomyces venezuelae*. J. Biol. Chem..

[115] Pfeiffer M., Johansson C., Krojer T., Kavanagh K.L., Oppermann U., Nidetzky B. (2019). A parsimonious mechanism of sugar dehydration by human GDP-mannose-4,6-dehydratase. ACS Catal..

[116] Creuzenet C., Lam J.S. (2001). Topological and functional characterization of WbpM, an inner membrane UDP-GlcNAc C6 dehydratase essential for lipopolysaccharide biosynthesis in *Pseudomonas aeruginosa*. Mol. Microbiol..

[117] Li Z., Hwang S., Ericson J., Bowler K., Bar-Peled M. (2015). Pen and pal are nucleotide-sugar dehydratases that convert UDP-GlcNAc to UDP-6-deoxy-d-GlcNAc-5,6-ene and then to UDP-4-keto-6-deoxy-L-AltNAc for CMP-pseudaminic acid synthesis in *Bacillus thuringiensis*. J. Biol. Chem..

[118] Zolghadr B., Gasselhuber B., Windwarder M., Pabst M., Kracher D., Kerndl M., Zayni S., Hofinger-Horvath A., Ludwig R., Haltrich D., Oostenbrink C., Obinger C., Kosma P., Messner P., Schäffer C. (2015). UDP-sulfoquinovose formation by *Sulfolobus acidocaldarius*. Extremophiles.

[119] Meyer B.H., Zolghadr B., Peyfoon E., Pabst M., Panico M., Morris H.R., Haslam S.M., Messner P., Schäffer C., Dell A., Albers S.V. (2011). Sulfoquinovose synthase - an important enzyme in the *N*-glycosylation pathway of *Sulfolobus acidocaldarius*. Mol. Microbiol..

[120] Mulichak A.M., Theisen M.J., Essigmann B., Benning C., Garavito R.M. (1999). Crystal structure of SQD1, an enzyme involved in the biosynthesis of the plant sulfolipid headgroup donor UDP-sulfoquinovose. Proc. Natl. Acad. Sci. U. S. A..

[121] Hong L., Zhao Z., Melançon C.E., Zhang H., Liu H.W. (2008). *In vitro* characterization of the enzymes involved in TDP-D-forosamine biosynthesis in the spinosyn pathway of *Saccharopolyspora spinosa*. J. Am. Chem. Soc..

[122] Bessman M.J., Frick D.N., O'Handley S.F. (1996). The MutT proteins or “Nudix” hydrolases, a family of versatile, widely distributed, “housecleaning” enzymes. J. Biol. Chem..

[123] He X.M., Liu H.W. (2002). Formation of unusual sugars: Mechanistic studies and biosynthetic applications. Annu. Rev. Biochem..

[124] Smith P., Lin A., Szu P.H., Liu H.W., Tsai S.C. (2006). Biosynthesis of a 3,6-dideoxyhexose: Crystallization and X-ray diffraction of CDP-6-deoxy-L-threo-D-glycero-4-hexulose-3-dehydrase (E1) for ascarylose biosynthesis. Acta Crystallogr. Sect. F Struct. Biol. Cryst. Commun..

[125] Cook P.D. (2006). The structure of GDP-4-keto-6-deoxy-d-mannose-3-dehydratase: A unique coenzyme B6-dependent enzyme. Protein Sci..

[126] Agnihotri G., Liu Y., Paschal B.M., Liu H. (2004). Identification of an unusual [2Fe-2S]-binding motif in the CDP-6-deoxy-D-glycero-L-threo-4-hexulose-3-dehydrase from *Yersinia pseudotuberculosis*: Implication for C-3 deoxygenation in the biosynthesis of 3,6-dideoxyhexoses. Biochemistry.

[127] Wu Q., Liu Y.N., Chen H., Molitor E.J., Liu H.W. (2007). A retro-evolution study of CDP-6-deoxy-d-glycero-L-threo-4-hexulose-3-dehydrase (E1) from *Yersinia pseudotuberculosis*: Implications for C-3 deoxygenation in the biosynthesis of 3,6-dideoxyhexoses. Biochemistry.

[128] Thoden J.B., Frey P.A., Holden H.M. (1996). Molecular structure of the NADH/UDP-glucose abortive complex of UDP-galactose 4-epimerase from Escherichia coli: Implications for the catalytic mechanism. Biochemistry.

[129] Beerens K., Soetaert W., Desmet T. (2012). Characterization and mutational analysis of the UDP-Glc(NAc) 4-epimerase from *Marinithermus hydrothermalis*. Appl. Microbiol. Biotechnol..

[130] Ferek J.D., Thoden J.B., Holden H.M. (2020). Biochemical analysis of a sugar 4,6-dehydratase from *Acanthamoeba polyphaga Mimivirus*. Protein Sci..

[131] Koropatkin N.M., Holden H.M. (2005). Structure of CDP-d-glucose 4,6-dehydratase from *Salmonella typhi* complexed with CDP-d-xylose. Acta Crystallogr. D Biol. Crystallogr..

[132] Vogan E.M., Bellamacina C., He X., Liu H., Ringe D., Petsko G.A. (2004). Crystal structure at 1.8 Å resolution of CDP-d-glucose 4,6-dehydratase from *Yersinia pseudotuberculosis*. Biochemistry.

[133] Cho A.R., Lee S.J., Kim B.G., Ahn J.-H. (2016). Biosynthesis of three N-acetylaminosugar-conjugated flavonoids using engineered Escherichia coli. Microb. Cell Fact..

[134] Ishiyama N., Creuzenet C., Lam J.S., Berghuis A.M. (2004). Crystal structure of WbpP, a genuine UDP-N-acetylglucosamine 4-epimerase from Pseudomonas aeruginosa. J. Biol. Chem..

[135] Creuzenet C., Schur M.J., Li J., Wakarchuk W.W., Lam J.S. (2000). FlaA1, a new bifunctional UDP-GlcNAc C6Dehydratase/C4 reductase from Helicobacter pylori. J. Biol. Chem..

[136] Creuzenet C. (2004). Characterization of CJ1293, a new UDP-GlcNAc C 6 dehydratase from *Campylobacter jejuni*. FEBS Lett..

